# A Multifaceted Implementation Strategy to Increase Out-of-Office Blood Pressure Monitoring: The EMBRACE Cluster Randomized Clinical Trial

**DOI:** 10.1001/jamanetworkopen.2023.34646

**Published:** 2023-09-25

**Authors:** Ian M. Kronish, Erica Phillips, Carmela Alcántara, Eileen Carter, Joseph E. Schwartz, Daichi Shimbo, Maria Serafini, Rebekah Boyd, Melinda Chang, Xiaohui Wang, Dominic Razon, Akash Patel, Nathalie Moise

**Affiliations:** 1 Center for Behavioral Cardiovascular Health, Columbia University Irving Medical Center, New York, New York; 2 Division of General Internal Medicine, Weill Cornell Medicine, New York, New York; 3 School of Social Work, Columbia University, New York, New York; 4 School of Nursing, University of Connecticut, Storrs; 5 Department of Psychiatry and Behavioral Health, Renaissance School of Medicine, Stony Brook University, Stony Brook, New York; 6 Division of Cardiology, Columbia University Irving Medical Center, New York, New York; 7 Department of Surgery, Duke University School of Medicine, Durham, North Carolina

## Abstract

**Importance:**

Few primary care patients complete guideline-recommended out-of-office blood pressure (BP) monitoring prior to having hypertension diagnosed.

**Objective:**

To evaluate the effectiveness of a behavioral theory–informed, multifaceted implementation strategy on out-of-office BP monitoring (ambulatory BP monitoring [ABPM] or home BP monitoring [HBPM]) among patients with new hypertension.

**Design, Setting, and Participants:**

This 2-group, pre-post cluster randomized trial was conducted within a primary care network of 8 practices (4 intervention practices with 99 clinicians; 4 control practices with 55 clinicians) and 1186 patients (857 intervention; 329 control) with at least 1 visit with elevated office BP and no prior hypertension diagnosis between October 2016 and September 2017 (preimplementation period) or between April 2018 and March 2019 (postimplementation period). Data were analyzed from February to July 2023.

**Interventions:**

Usual care (control group) or a multifaceted implementation strategy consisting of an accessible ABPM service; electronic health record (EHR) tools to facilitate test ordering; clinician education, reminders, and feedback relevant to out-of-office BP monitoring; nurse training on HBPM; and patient information handouts.

**Main Outcomes and Measures:**

The primary outcome was patient completion of out-of-office BP monitoring within 6 months of an eligible visit. Secondary outcomes included clinician ordering of out-of-office BP monitoring. Blinded assessors extracted outcomes from the EHR.

**Results:**

A total of 1186 patients (857 intervention; 329 control) were included, with a mean (SD) age of 54 (16) years; 808 (68%) were female, and 549 (48%) were Spanish speaking; among those with race and ethnicity documented, 123 (10%) were Black or African American, and 368 (31%) were Hispanic. Among intervention practices, the percentage of visits resulting in completed out-of-office BP monitoring increased from 0.6% (0% ABPM; 0.6% HBPM) to 5.7% (3.7% ABPM; 2.0% HBPM) between the preimplementation and postimplementation periods (*P* = .009). Among control practices, the percentage of visits resulting in completed out-of-office BP monitoring changed from 5.4% (0% ABPM; 5.4% HBPM) to 4.3% (0% ABPM; 4.3% HBPM) during the corresponding period (*P* = .94). The ratio of relative risks (RRs) of out-of-office BP monitoring in the postimplementation vs preimplementation periods for intervention vs control practices was 10.5 (95% CI, 1.9-58.0; *P* = .01). The ratio of RRs of out-of-office BP monitoring being ordered was 2.2 (95% CI, 0.8-6.3; *P* = .12).

**Conclusions and Relevance:**

This study found that a theory-informed implementation strategy that included access to ABPM modestly increased out-of-office BP monitoring among patients with elevated office BP but no hypertension diagnosis.

**Trial Registration:**

ClinicalTrials.gov Identifier: NCT03480217

## Key Points

**Question:**

Is a theory-informed multifaceted implementation strategy that includes access to an ambulatory blood pressure (BP) monitoring service effective at increasing out-of-office BP monitoring among primary care patients with elevated office BP in accordance with US hypertension screening guidelines?

**Findings:**

In this cluster randomized trial including 8 safety-net practices and 1186 patients with elevated office BP but no hypertension diagnosis, the implementation strategy modestly increased patient completion of out-of-office BP monitoring.

**Meaning:**

These findings suggest that there is a need for more intensive implementation strategies for increasing adherence to hypertension screening guidelines that recommend out-of-office BP monitoring before hypertension diagnosis.

## Teaser

This cluster randomized trial evaluates the effectiveness of a behavioral theory–informed, multifaceted implementation strategy on out-of-office blood pressure monitoring among primary care patients with elevated office BP but no prior diagnosis of hypertension.

## Introduction

Approximately 20% of patients with elevated office blood pressure (BP) readings but no prior diagnosis of hypertension do not have elevated readings when BP is measured out of the office.^1,2^ This mismatch in BP is commonly referred to as white-coat hypertension.^3^ Evidence suggests that white-coat hypertension does not confer substantial cardiovascular risk.^2,4,5,6^ Thus, failure to identify white-coat hypertension can lead to inappropriate labeling of patients with a chronic disease and overtreatment with medications.^7,8,9,10^ Based on these observations, the US Preventive Services Task Force updated their hypertension screening recommendations in 2015 to advise that patients with elevated office BP undergo out-of-office BP monitoring with either ambulatory BP monitoring (ABPM)^11,12^ or home BP monitoring (HBPM)^13^ to exclude white-coat hypertension prior to a diagnosis of hypertension.

Despite the widespread availability of HBPM devices and reimbursement for ABPM, both are infrequently utilized as part of hypertension screening in the United States.^14,15,16,17^ To our knowledge, there have been few if any interventions designed to increase the use of out-of-office BP monitoring in this context. Developing an effective and sustainable model for increasing the use of guideline-recommended out-of-office BP monitoring is well-suited to the emerging area of implementation science, which involves the study of methods to increase the uptake of evidence-based interventions.^18^

In the current study, we used the Behavior Change Wheel (BCW)^19^ to develop a multifaceted implementation strategy that addresses barriers to out-of-office BP monitoring as part of hypertension screening. The BCW is a multistep framework increasingly used to design implementation strategies.^20,21,22^ It involves first mapping barriers with stakeholder input and then identifying behavioral theory–informed intervention components for addressing these barriers. We then evaluated the effect of this implementation strategy on out-of-office BP monitoring in patients with elevated office BP but no prior diagnosis of hypertension.

## Methods

### Study Design

Details of the study design and protocol for the Effects of a Multifaceted Intervention on Blood Pressure Actions in the Primary Care Environment (EMBRACE) trial were previously described.^23^ Briefly, EMBRACE was a pre-post cluster randomized trial with randomization at the practice level for practical reasons relevant to clinical implementation and to prevent contamination between clinicians within the same practice. The study protocol (Supplement 1) was approved by the institutional review boards of Columbia University Irving Medical Center (CUIMC) and Weill Cornell Medicine (WCM). Requirements to obtain informed consent from patients and clinicians were waived as participation was deemed minimal risk. The Consolidated Standards of Reporting Trials extension (CONSORT Extension) to cluster randomized trials reporting guideline was followed.^24^

### Study Setting

The trial was conducted in primary care clinics that are part of the Ambulatory Care Network (ACN) of New York-Presbyterian (NYP), a health enterprise affiliated with 2 medical schools, CUIMC and WCM. The preimplementation period took place from October 1, 2016, to September 30, 2017; implementation from October 1, 2017, to March 31, 2018; and postimplementation from April 1, 2018, to March 31, 2019. The ACN serves a predominantly low-income, publicly insured population with substantial numbers of Hispanic and Black or African American patients. The 10 primary care practices in the ACN that serve adult patients, each in geographically distinct locations, are staffed by internal medicine physicians, family medicine physicians, nurse practitioners, and graduate medical education trainees. Office BP was taken by medical assistants using automated devices, with readings manually entered into the electronic health record (EHR). None of the practices had protocols for systematically repeating elevated office BP readings. The practices used 2 different EHR systems: Allscripts (Allscripts) at CUIMC-affiliated practices and Epic (Epic) at WCM-affiliated practices.

### Eligibility

Primary care practices that served adult patients, were part of the ACN, and whose medical directors agreed to participate were included. Of 10 such practices, the 2 used for pilot testing the implementation strategy were excluded. Patients were identified through the EHR and were included if they were 18 years old or older, had not previously been diagnosed with hypertension, and had high office BP (systolic BP ≥140 mm Hg or diastolic BP ≥90 mm Hg) during at least 1 scheduled visit with a primary care clinician at an eligible practice during the relevant time period, hereafter referred to as eligible visits. If multiple BP readings were documented in the flowsheet, the mean was used to determine eligibility. Patients were excluded if they had a prior diagnosis of hypertension; a prior evaluation for white-coat hypertension; prior prescribed antihypertensive medication; office BP less than 140/90 mm Hg; severely elevated office BP (systolic BP ≥180 mm Hg or diastolic BP ≥110 mm Hg); or evidence of target organ damage (chronic kidney disease with creatinine levels >1.5 mg/dL [to convert to millimoles per liter, multiply by 88.4], prior history of stroke, transient ischemic attack, coronary artery disease, myocardial infarction, congestive heart failure, or peripheral artery disease). As each visit at which patients had elevated office BP represented an opportunity to order out-of-office BP monitoring, patients could be eligible at more than 1 visit.

### Recruitment, Randomization, and Allocation

The study principal investigator (I.M.K.) met with the medical directors of all 8 potentially eligible practices to invite their practice to participate, and each agreed. The 8 practices were then matched in pairs according to practice size and patient and clinician characteristics (ie, 2 practices that care for people living with HIV; 2 smaller internal medicine practices without trainees; 2 larger internal medicine practices with trainees; and 2 remaining practices—family medicine and geriatrics—without obvious pairings). Randomization was generated by the study’s (blinded) biostatistician (J.E.S.), using a random number generator to assign one practice within each matched pair to the intervention.

### Intervention and Control Conditions

Details on the development of the implementation strategy using the BCW have previously been published.^23^ Briefly, patients and primary care clinicians were interviewed to understand barriers to implementation (eg, lack of accessible ABPM service).^25,26^ The multidisciplinary implementation strategy design team then used the BCW to develop a preliminary implementation strategy. Medical directors and ACN leaders were then interviewed to determine which implementation strategy components should be retained or added and which should be rejected by virtue of their not meeting one or more APEASE (acceptability, practicability, effectiveness, affordability, side-effects, and equity) criteria.^27^ The final planned strategy for increasing the completion of ABPM/HBPM consisted of a multilevel intervention including (1) access to ABPM; (2) EHR tools tailored to distinct EHR capabilities at WCM- and CUIMC-affiliated practices to facilitate test ordering; (3) educational presentations on why, when, and how to order out-of-office BP monitoring; (4) feedback on the utilization and results of ABPM; (5) reminders to order out-of-office BP monitoring; (6) nurse training on how to teach HBPM to patients, and (7) patient information handouts (eTable 1 in Supplement 2).

Practices randomized to the usual care control condition continued to screen for hypertension according to their usual practice without the benefit of the implementation strategy. Patients from these practices could be referred for ABPM by their clinicians, but no special education, training, or EHR support was provided to promote ABPM, and as the ABPM services were new, there was little clinician awareness of these services at usual care practices.

### Study Outcomes

The primary outcome was completion of ABPM or HBPM within 6 months of an eligible visit. Two medically trained abstractors blinded to group assignment independently reviewed the EHR for evidence of ABPM or HBPM completion. Discrepancies were resolved through consensus with a third study team member. ABPM was coded as complete if 10 or more awake BP readings were available.^28^ HBPM was coded as complete if there was mention of patients having a log of home BP readings in subsequent office visit notes. BP readings obtained at a pharmacy or kiosk were not eligible. The prespecified secondary outcome was clinician ordering of ABPM or HBPM at the time of an eligible visit. Other possible clinician actions at eligible visits included diagnosing hypertension or taking no action as evidenced by no mention of BP in the assessment and plan section of the office visit note or BP mentioned but no action taken pertaining to diagnosing or treating hypertension.

### Statistical Analysis

The percentage of eligible patient visits resulting in ABPM and HBPM being completed in the preimplementation and postimplementation periods was calculated for intervention and control clinics, separately. When reporting condition-by-period rates, simple percentages with 95% CIs were used. Multilevel Poisson regression models,^29,30^ where level 1 was an eligible patient visit and level 2 was the practice, were used to test whether the preintervention to postintervention change in the rate of out-of-office BP completion (primary outcome) was greater in the practices that received the intervention than in the control practices. The same approach was used to evaluate the effect of the intervention on the rate of out-of-office BP ordering. Relative risks (or risk ratios) (RRs) and 95% CIs were based on multilevel Poisson regression analyses adjusted for within-practice clustering. Sensitivity analyses were conducted adjusting for patient age and sex.

We estimated the power to detect a 10% increase in out-of-office BP completion rate due to the intervention (ie, RR 3.0; 15% vs 5%; at a 2-tailed, α = .05 significance level, accounting for within-practice clustering (equivalently, between-practice variability). For a multilevel Poisson regression model with log link function, this variability depends on the coefficient of variation (CV; the SD of practice-level out-of-office BP completion rates divided by their mean).^31^ For each combination of CV (0 to 0.40, in increments of 0.10) and correlation (*r*) between preintervention and postintervention practice-level out-of-office BP completion rates (0.50 to 0.90, in increments of 0.10), the power to detect an RR of 3.0 was estimated by (1) generating 10 000 simulated data sets, incorporating the number of eligible patient visits during 2014 in each of the 8 participating practices; (2) performing the multilevel Poisson regression analysis on each simulated data set, and (3) determining the proportion of data sets in which the null hypothesis was rejected (2-tailed, α = .05) in the hypothesized direction. According to these simulations, the study would have 84% power to detect the hypothesized condition × period interaction effect (primary analysis) if there was no between-practice heterogeneity; 79% to 80% power if CV was 0.10 and *r* 0.50 or greater, and 75% power if CV was 0.40 and *r* 0.90.^23^ Data analysis was conducted with SAS version 9.4 (SAS Institute) from February to July 2023.

## Results

**Figure 1.**
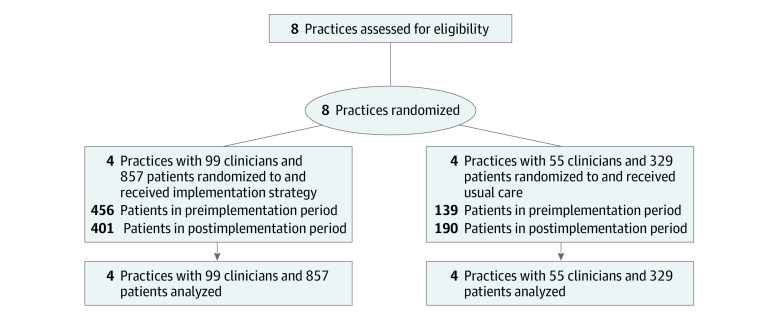
Recruitment and Randomization of Primary Care Practices in the Effects of a Multifaceted Intervention on Blood Pressure Actions in the Primary Care Environment (EMBRACE) Cluster Randomized Trial

A total of 1186 patients (857 intervention; 329 control) were included, with a mean (SD) age of 54 (16) years, 808 (68%) were female, and 549 (48%) had Spanish as their preferred language. Among those with race and ethnicity documented, 123 (10%) were Black or African American, and 368 (31%) were Hispanic. These patients were treated by 154 clinicians and had 1339 eligible visits (Figure 1). Characteristics of clinicians and patients were similar in the preimplementation and postimplementation periods (Table 1; eTable 2 in Supplement 2). Despite matching practices before randomization, there were substantial differences in the number and characteristics of clinicians and patients between intervention and control practices. Patients in control practices were older and a greater percentage were female, White, and had dementia. Control practices included a greater proportion of attendings than trainees. Geriatricians were primarily clustered in control practices, whereas family medicine physicians were only located in intervention practices.

**Table 1.** Patient Characteristics According to Group Assignment^a^

Characteristics	Patients, No. (%)			
	Intervention		Usual care	
	Preimplementation (n = 456)	Postimplementation (n = 401)	Preimplementation (n = 139)	Postimplementation (n = 190)
Age, mean (SD), y	51.6 (14.1)	51.3 (14.5)	58.9 (16.0)	59.8 (19.1)
Sex				
Female	289 (63.4)	257 (64.1)	105 (75.5)	157 (82.6)
Male	167 (36.6)	144 (35.9)	34 (24.5)	33 (17.4)
Ethnicity				
Hispanic	152 (33.3)	113 (28.2)	50 (36.0)	53 (27.9)
Non-Hispanic	70 (15.4)	79 (19.7)	17 (12.2)	43 (22.6)
Not reported	234 (51.3)	209 (52.1)	72 (51.8)	94 (49.5)
Race^b^				
Black/African American	53 (11.6)	50 (12.5)	9 (6.5)	11 (5.8)
White	86 (18.9)	69 (17.2)	33 (23.7)	57 (30.0)
Other	29 (6.4)	8 (2.0)	6 (4.3)	6 (3.2)
Not reported	288 (63.2)	274 68.3	91 (65.5)	116 (61.1)
Preferred language				
English	193 (43.7)	205 (52.6)	64 (47.4)	85 (46.2)
Spanish	230 (52.0)	163 (41.8)	70 (51.9)	86 (46.7)
Other	19 (4.3)	22 (5.6)	1 (0.7)	13 (6.1)
Systolic BP, mean (SD), mm Hg	144.7 (9.2)	144.1 (8.4)	145.1 (9.2)	143.3 (10.1)
Diastolic BP, mean (SD), mm Hg	86.3 (7.6)	85.9 (8.0)	82.9 (10.1)	83.4 (8.7)
Diabetes	51 (11.3)	55 (13.9)	23 (16.9)	24 (12.8)
Dementia	1 (0.2)	5 (1.3)	9 (6.6)	8 (4.3)
Depression	115 (25.4)	117 (29.5)	38 (27.7)	58 (30.7)
Smoking	60 (13.3)	48 (12.1)	12 (8.8)	16 (8.5)
No. of medications, median (IQR)	3 (1-5)	3 (1-6)	3 (1-6)	3 (1-7)

Abbreviation: BP, blood pressure.

^a^ Fewer than 3% of responses were missing for all categories other than ethnicity and race.

^b^ All patient characteristics including race and ethnicity were extracted from the electronic health record. Other race included Native Hawaiian or Other Pacific Islander (n = 17 total), Asian (n = 10), American Indian or Alaska Native (n = 1), and “more than one race” (n = 21).

**Figure 2.**
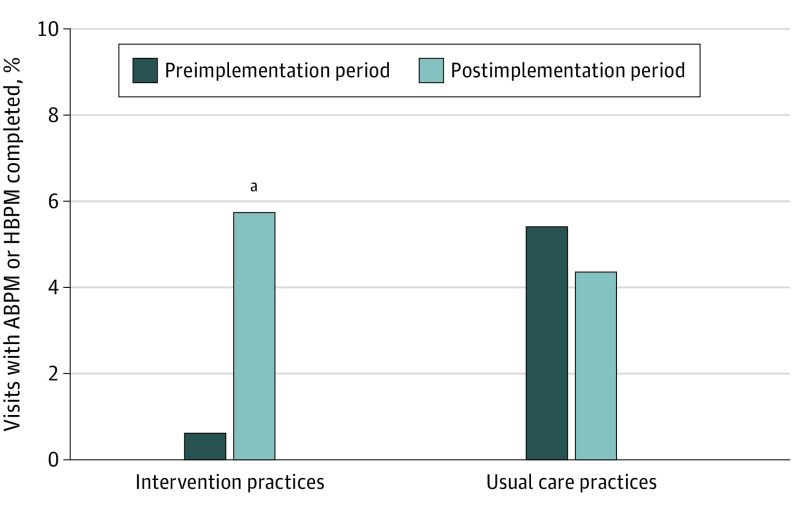
Percentage of Eligible Patient Visits That Resulted in Completed Ambulatory Blood Pressure Monitoring (ABPM) or Home Blood Pressure Monitoring (HBPM) in the 12-Month Preimplementation vs 12-Month Postimplementation Periods The study was conducted at 8 practices with 1186 patients and 1339 eligible patient visits. Visits were considered eligible if patient had elevated office blood pressure and no prior diagnosis of hypertension. ^a^*P* < .009

All components of the implementation strategy were delivered to each practice allocated to the intervention. Among intervention practices, completion of out-of-office BP monitoring increased from 0.6% of eligible visits (3 of 529; 0% ABPM; 0.6% HBPM) to 5.7% of eligible visits (26 of 454; 3.7% ABPM [17 visits]; 2.0% [9 visits] HBPM) between the preimplementation and postimplementation periods (RR, 10.08; 95% CI, 2.26-45.00; *P* = .009) (Tables 2 and 3 and Figure 2). Among control practices, completion of out-of-office BP monitoring changed from 5.4% of visits (8 of 149; 0% ABPM; 5.4% HBPM) to 4.3% of visits (9 of 207; 0% ABPM; 4.3% HBPM) of visits between the preimplementation and postimplementation periods (RR, 0.96; 95% CI, 0.29-3.20; *P* = .94). Overall, the ratio of these RRs (the prespecified primary parameter of interest) was 10.49 (95% CI, 1.90-58.01; *P* = .01). The same pattern of results was present in analyses adjusted for patient age and sex (eTable 3 in Supplement 2).

**Table 2.** Visits at Which Out-of-Office Blood Pressure Monitoring Was Ordered and Completed in Preimplementation and Postimplementation Periods According to Group Assignment

Outcome	Visits, No. (%)^a^			
	Intervention (n = 4 practices)		Usual care (n = 4 practices)	
	Preimplementation (n = 529 visits)	Postimplementation (n = 454 visits)	Preimplementation (n = 149 visits)	Postimplementation (n = 207 visits)
ABPM or HBPM testing completed by patient	3 (0.6)	26 (5.7)	8 (5.4)	9 (4.3)
HBPM completed by patient	3 (0.6)	9 (2.0)	8 (5.4)	9 (4.3)
ABPM completed by patient	0	17 (3.7)	0	0
ABPM or HBPM ordered by clinician	15 (2.8)	36 (7.9)	13 (8.7)	19 (9.2)
HBPM ordered by clinician	15 (2.8)	14 (3.1)	13 (8.7)	19 (9.2)
ABPM ordered by clinician	0	22 (4.8)	0	0

Abbreviations: ABPM, ambulatory blood pressure monitoring; HBPM, home blood pressure monitoring.

^a^ Visits refers to the number of scheduled primary care visits at which patients had elevated office blood pressure and no prior diagnosis of hypertension.

**Table 3.** Relative Risks of Ordering and Completing Out-of-Office Blood Pressure Monitoring in the Postimplementation vs Preimplementation Periods

Outcome	Postimplmentation vs preimplementation, intervention	*P* value	Postimplementation vs preimplementation, usual care	*P* value	Postimplementation vs preimplementation, intervention vs usual care	*P* value	Between practice variability (CV)
ABPM or HBPM completed by patient	10.08 (2.26-45.00)	.009	0.96 (0.29-3.20)	.94	10.49 (1.90-58.01)	.01	0.65
HBPM completed by patient	3.40 (0.66-17.61)	.12	0.95 (0.28-3.17)	.92	3.59 (0.58-22.18)	.15	0.56
ABPM completed by patient^a^	NA	NA	NA	NA	NA	NA	NA
ABPM or HBPM ordered by clinician	2.77 (1.30-5.91)	.02	1.25 (0.51-3.04)	.56	2.22 (0.78-6.28)	.12	0.69
HBPM ordered by clinician	1.05 (0.42-2.63)	.90	1.24 (0.51-3.02)	.58	0.85 (0.27-2.65)	.76	0.59
ABPM ordered by clinician^a^	NA	NA	NA	NA	NA	NA	NA

Abbreviations: ABPM, ambulatory blood pressure monitoring; CV, coefficient of variation; HBPM, home blood pressure monitoring; NA, not applicable.

^a^ Not analyzed, as no ABPM completed or ordered in preimplementation periods in either arm nor in the post implementation in the usual care arm.

Among intervention practices, ordering of out-of-office BP monitoring increased from 15 of 529 visits (2.8%; 0% ABPM; 2.8% HBPM) to 36 of 454 visits (7.9%; 22 [4.8%] ABPM; 14 [3.1%] HBPM) between the preimplementation and postimplementation periods (RR, 2.77; 95% CI, 1.30-5.91; *P* = .02). Among control practices, out-of-office BP test ordering changed from 13 of 149 visits (8.7%; 0% ABPM; 8.7% HBPM) to 19 of 207 visits (9.2%; 0% ABPM; 9.2% HBPM) between the preimplementation and postimplementation periods (RR, 1.25; 95% CI, 0.51-3.04; *P* = .56). The ratio of these RRs was 2.22 (95% CI, 0.78-6.28; *P* = .12).

When out-of-office BP monitoring was not ordered, the predominant action was to wait-and-see until the next visit. Specifically, during the postimplementation period in intervention practices, after excluding visits at which out-of-office BP monitoring was ordered, no action was taken in 391 of 418 remaining visits (93.5%) and hypertension was diagnosed in 23 visits (5.5%). A similar pattern was found in control practices.

In post hoc analyses unadjusted for clustering by practice, of patients with ABPM ordered during the postimplementation period, all from intervention practices, ABPM was successfully completed in 17 of 22 patients (77.3%). Of patients with HBPM ordered, the percentage that completed HBPM increased from 3 of 15 (20.0%) preimplementation to 9 of 14 (64.3%) postimplementation in intervention practices (*P* = .03) while decreasing nonsignificantly from 8 of 13 patients (61.5%) to 9 of 19 patients (47.4%) (*P* = .49) in control practices.

Across both groups in both time periods, white-coat hypertension was diagnosed in 17 of 35 patients (48.6%) who completed out-of-office BP monitoring (4 of 14 [28.6%] ABPM; 13 of 21 [62.9%] HBPM). Too few patients completed out-of-office BP monitoring to compare differences in white-coat hypertension diagnosis between intervention and control practices.

## Discussion

In this cluster randomized trial, a theory-informed multifaceted implementation strategy targeting patient-, clinician-, and systems-level barriers increased out-of-office BP monitoring in patients with elevated office BP without a prior diagnosis of hypertension. These improvements were driven by increases in ABPM ordering and completion as well as increases in HBPM completion among those with HBPM ordered. In contrast, no patients had ABPM ordered or completed in usual care practices. These findings are consistent with the low use of ABPM in US-based primary care settings^32^ and support that increasing the accessibility of ABPM, paired with education and reminders, can increase uptake of ABPM in primary care, at least marginally. The value of out-of-office BP monitoring was confirmed, as approximately half of patients who completed out-of-office BP monitoring were diagnosed with white-coat hypertension and potentially avoided inappropriate treatment and chronic disease labeling.

Although the implementation strategy was effective and could be implemented with fidelity, it only modestly influenced the behavior of clinicians. After implementation, out-of-office BP monitoring was ordered in less than 10% of visits with guideline-eligible patients and completed in only 72% of patients that had testing ordered. Making ABPM available on-site at individual practices may have been a more potent approach. Indeed, training practices to provide ABPM was considered as a potential implementation strategy component, but this component was not viewed as feasible by key stakeholders due to resource limitations in terms of staff time to place, track, and download data from ABPM devices.^23^ Policy-level interventions relevant to billing and reimbursement are likely necessary to spur greater adoption of ABPM in this primary care network. Hard-stop EHR tools that required clinicians to consider out-of-office BP monitoring might also have prompted more action, yet this strategy was not viewed as acceptable in the primary care network where this study was conducted.

While the primary behavioral target for the implementation strategy was clinician ordering of out-of-office BP monitoring, the strategy included patient-focused components that may have increased the percentage of patients at intervention practices successfully completing HBPM from the preimplementation to the postimplementation periods. These components included clinician and nurse training on how to teach patients the correct HBPM protocol, EHR tools to facilitate prescribing of HBPM devices, and links to patient handouts that demonstrated their correct use. While HBPM ordering increased in intervention practices, the magnitude of improvement was small, perhaps due to system-level barriers unaddressed by the implementation strategy. These barriers included limited insurance coverage for home BP devices. In underresourced patient populations, out-of-pocket costs for HBPM devices may continue to serve as a barrier to HBPM testing and may thereby increase health disparities.^33^

The predominant action by clinicians for patients with elevated office BP readings was to wait and see. This contributes to the literature showing that hypertension is commonly underdiagnosed in primary care, leading to undertreatment.^34^ Future interventions should strive to reduce hypertension underdiagnosis in primary care.

### Limitations

There are several potential limitations of this study’s findings. Despite matching practices prior to randomization, there were substantial differences in the characteristics and numbers of patients and clinicians between intervention and control practices. This is more likely to happen when, as in this study, only a small number of practices are enrolled. There were also differences in preimplementation test ordering. Nevertheless, comparing the pre-post within-condition changes in out-of-office BP monitoring helped control for these differences and represented a strength of the pre-post cluster randomized design. While cluster randomization was applied at the practice level, some implementation strategy components may have been received by control clinicians; this would have biased results toward the null. The implementation strategy was tested in a small number of hospital-affiliated practices that served an underresourced patient population in one geographic region. Findings might not generalize to other settings. Strategies, such as home BP device loaner programs and increased staff training in provision of out-of-office BP monitoring, that were not viewed as feasible in this network may be effective elsewhere. There may have been differences in the extent to which clinicians in the intervention and control practices documented HBPM, such that differences in HBPM completion should be interpreted cautiously. Also, it was not possible to determine whether patients followed the correct HBPM protocol; patients in intervention practices may have received better training on the proper conduct of HBPM. Finally, just prior to the postimplementation period, the 2017 American College of Cardiology/American Heart Association BP guideline recommended lowering the threshold for diagnosing hypertension from 140/90 mm Hg to 130/80 mm Hg. Future studies should assess the impact of these guideline changes on hypertension screening and their implications for developing implementation strategies.

## Conclusions

This study found that a theory-informed implementation strategy developed based on the BCW modestly increased out-of-office BP monitoring in patients with elevated office BP and no prior diagnosis of hypertension. Patient-, clinician-, and system-level interventions, such as higher reimbursement and lower out-of-pocket costs for out-of-office BP monitoring, may be needed for greater uptake of hypertension screening guidelines.

## Notes

Supplement 1.
Trial Protocol and Statistical Analysis Plan

Supplement 2. **eTable 1.** Description of Implementation Strategy Components **eTable 2. **Clinician Characteristics According to Group Assignment **eTable 3.** Relative Risks (95% Confidence Interval) of Ordering and Completing Out-of-Office Blood Pressure Monitoring in the Post- vs Pre-Implementation Periods, Adjusting for Age and Sex

Supplement 3.
Data Sharing Statement
