# Supplementary material for: A Multifaceted Implementation Strategy to Increase Out-of-Office Blood Pressure Monitoring: The EMBRACE Cluster Randomized Clinical Trial
Source: JAMA Netw Open. 2023 Sep 25;6(9):e2334646. doi: 10.1001/jamanetworkopen.2023.34646 (PMC10520739; doi:10.1001/jamanetworkopen.2023.34646)
Supplement: Supplement 1. — Trial Protocol and Statistical Analysis Plan [file jamanetwopen-e2334646-s001.pdf]

Effects of a **M**ulti-faceted Intervention on **B**lood P**r**essure **A**ctions in the Primary **C**are **E**nvironment:
The EMBRACE Cluster Randomized Trial
ClinicalTrials.gov: NCT03480217

Study Protocol

Version 3

Last updated 5/10/2023

Table of Contents

Protocol Amendments……………………………………………………………..…4
Administrative Information 5
Title Registration Data 5
Introduction 8
Background and Rationale 8
Objectives/Hypothesis 10
Trial Design 10
Methods 11
Participant, Interventions and Outcomes 11
Study Setting: Coordinating Center 11
Study Setting: Recruitment Sites 11
Detailed Eligibility Criteria 11
Interventions 12
Outcomes 13
Participant Timeline 14
Sample Size 15
Recruitment 17
Assignment of Interventions 17
Sequence generation 18
Concealment Mechanism 18
Implementation 18
Blinding 18
Data Collection, Management and Analysis 19
Data Collection Methods 19
Data Management 19
Statistical Methods 20
Data Monitoring 22
Harms 22
Auditing 22
Ethics and Dissemination 23
Research Ethics Approval 23
Protocol Amendments 23
Informed Consent 23

Confidentiality 24
Declaration of Interests 24
Access to data 24
Post-trial care... 24
Dissemination policy 24

Protocol Amendments

Summary of changes

January 2021 — Unable to collect data on maintenance period due to changes in electronic health record with Epic implementation and staffing limitations due to Covid pandemic.

March 2023: — Reporting of the arms and outcomes was revised on Clinicaltrials.gov for clarity purposes.

Arms were increased from 2 to 4 so as to be able to present the pre- and post-implementation periods for the intervention and control conditions separately.

To reflect the statistical analysis plan that was published prior to the conduct of any analyses (Moise N, Phillips E, Carter E, et al. Design and study protocol for a cluster randomized trial of a multi-faceted implementation strategy to increase the uptake of the USPSTF hypertension screening recommendations: the EMBRACE study. Implement Sci. 2020;15:16.), the outcome reporting was changed from presenting outcomes as pre-post changes to presenting outcomes separately for intervention and control practices in the pre-implementation and post-implementation period. The statistical analysis plan follows the pre-planned analysis plan.

August 2023: — The description of the hypotheses was revised to indicate that the primary analyses compared the change in outcomes from the pre- to post-implementation periods in intervention versus control practices. This was done to increase consistency with the statistical analysis plan.

Within the statistical section, clarified that the primary outcome, completion of out-of-office BP monitoring, had to be completed within 6 months of eligible visits. This was previously specified elsewhere.

Administrative Information

Title Registration Data

**Scientific Title**:	**E**ffects of a **M**ulti-faceted Intervention on **B**lood P**r**essure **A**ctions in the Primary **C**are **E**nvironment: The EMBRACE Cluster Randomized Trial (Acronym: EMBRACE)
**Public Title**:	**E**ffects of a **M**ulti-faceted Intervention on **B**lood P**r**essure **A**ctions in the Primary **C**are **E**nvironment: The EMBRACE Cluster Randomized Trial
**Trial Registration**:	ClinicalTrials.gov: NCT03480217
**Secondary Identifiers**:	Columbia University Irving Medical Center: IRB#AAAQ1062<br>Weill Cornell Medicine: IRB# 1701017937
**Funding Agency**:	Agency for Healthcare Research and Quality (AHRQ)<br>Application Number: R01 HS024262
**Primary Sponsor**:	Columbia University
**Collaborators:**	Weill Cornell Medicine
**Contact for Scientific or Public Queries**:	Ian M. Kronish, MD, MPH, Principal Investigator<br>Associate Professor of Medicine<br>Associate Director, Center for Behavioral Cardiovascular Health<br>Co-Director, Columbia Hypertension Center<br>Department of Medicine<br>Columbia University Irving Medical Center<br>622 West 168th Street, PH9-311<br>New York, NY 10032<br>212.342.1335<br>ik2293@columbia.edu
**Countries of Recruitment**:	USA only
**Health Condition(s) or problem(s) studied**:	Hypertension, screening, diagnosis, white-coat
**Interventions:**	Randomization of practices to a multi-faceted implementation strategy intervention versus usual care control
**Key Eligibility Criteria*:**	

Practices

Inclusion criteria:

- Primary care practice in New York Presbyterian Hospital's Ambulatory Care Network that provide care to adult patients

Exclusion criteria:

- Medical director declines to participate in trial
- Site for pilot testing the intervention

Clinicians

Inclusion Criteria:

- Primary care clinician that provides scheduled primary care visits with adult patients

Exclusion Criteria:

- None

Patients

Inclusion Criteria

- Adult patient (18 years or older) with scheduled primary care visit at eligible practice
- Elevated office BP (BP ≥ 140/90 mmHg) without a prior diagnosis of hypertension

Exclusion Criteria:

- Prior diagnosis of white coat hypertension
- Prior evaluation for white-coat hypertension by 24-hr ABPM or HBPM
- Prior prescribed antihypertensive medication
- Manual office BP <140/90 mmHg
- Severely elevated office BP (systolic BP ≥ 180 mmHg or diastolic BP ≥ 110 mmHg)
- Evidence of target-organ damage (chronic kidney disease with creatinine > 1.5 mg/dL or prior history of stroke, transient ischemic attack, coronary artery disease, myocardial infarction, congestive heart failure, or peripheral arterial disease) as per electronic health record review by a medically trained chart abstractor

Study Type: 2-arm parallel group cluster randomized trial

Date of First Enrollment: April 1, 2018

Target Sample Size: 8 practices, 138 primary care clinicians

Recruitment Status: Completed recruitment

Primary Outcomes: Percentage of visits at which guideline-eligible patients complete out-of-office BP monitoring, either ABPM or HBPM, within 6 months of an eligible visit

Key Secondary Outcomes:

- Percentage of visits at which out-of-office BP monitoring is ordered, either ABPM or HBPM,

MOP Version: 4 (Last updated: 8/12/2023)

Funding: AHRQ R01 HS024262

Introduction

Background and Rationale

The accurate diagnosis of hypertension is essential for targeting appropriate therapy at the patients who can most benefit from hypertension treatment. Inappropriate or overdiagnosis of hypertension can lead to unnecessary treatment with blood pressure (BP) medications, wasteful healthcare utilization, and adverse psychological consequences from labeling with a chronic disease.[1-4] There are challenges with measuring BP in the office that make overdiagnosis common. A systematic review found that 5%-65% of patients with elevated office BP do not have high out-of-office BP readings according to home BP monitoring (HBPM) or 24-hour ambulatory BP monitoring (ABPM).[5,6] This is commonly referred to as white-coat hypertension.[7] In contrast to patients with sustained hypertension (elevated BP in office and ambulatory settings), white-coat hypertension appears to confer little to no increased cardiovascular risk.[5,8] Based primarily on these observations, the USPSTF updated its hypertension screening guidelines in 2015 to recommended that patients with elevated office BP undergo ABPM or HBPM to rule-out white-coat hypertension prior to a new diagnosis of hypertension.[9] The 2017 American College of Cardiology and American Heart Association BP guidelines had similar recommendations regarding the use of out-of-office BP monitoring as part of hypertension diagnosis.[10]

Despite these guidelines, ABPM and HBPM are infrequently utilized as part of hypertension diagnosis in the U.S. In a study of Medicare beneficiaries, less than 0.1% were receiving ABPM, and those few who were billed for ABPM testing were in patients already treated with antihypertensive medications.[11,12] Several clinician-level barriers to ABPM have been proposed to explain the underuse of this evidence-based diagnostic test including lack of knowledge about the guideline and poor accessibility of ABPM, particularly in resource-poor practice settings.[13,14] Patient-level barriers have also been proposed, such as perceived discomfort of ABPM testing and disagreement with the need for testing.[15] However, there has not yet been any rigorous U.S.-based study of the barriers and facilitators to ABPM for diagnosing hypertension, nor have there been any studies of interventions to increase the use of ABPM prior to a hypertension diagnosis. While there is greater use of HBPM in the context of managing hypertension, it is inconsistently used to exclude white-coat hypertension before hypertension diagnosis. A recent study examining the use of HBPM in patients with elevated office BP but no diagnosis of hypertension found that only 4% were recommended to use HBPM by their clinicians in this setting and only 14% had ever used HBPM.[16] Barriers to HBPM in the context of hypertension screening were not well characterized, particularly in underserved US-based primary care patient populations.

Of note, in the update to the USPSTF hypertension screening recommendations finalized in 2021, masked hypertension was considered as another potential indication for out-of-office BP monitoring as part of hypertension screening.[17] Ultimately, the panel

determined that there was insufficient evidence to support screening for masked hypertension, but more research was needed, and masked hypertension screening could be recommended in future years. Accordingly, we conducted additional focus groups with primary care clinicians to understand clinician barriers to screening for masked hypertension, but did not use these data to inform our implementation strategy.

We endeavored to develop a theory-informed, scalable implementation strategy to address barriers to increasing the uptake of the recent USPSTF hypertension recommendation, which represents a paradigm shift in the diagnosis of hypertension (i.e., move from relying on office BP to out-of-office testing). The **setting** for this work was patients and clinicians in a primary care network that serves vulnerable patients in an urban setting. To develop a theory-informed strategy, we drew primarily on Michie and colleagues' BCW framework.[18] We chose this framework because it links identified behavioral targets to intervention functions most likely to bring about practice and clinician level change, and it has increasingly been used to develop implementation strategies.[19] We employed a multi-disciplinary stakeholder process to operationalize this multi-step process and developed a multi-component implementation strategy for increasing the completion of both ABPM and HBPM testing for the purposes of hypertension diagnosis. The BCW framework first prompts one to identify both a primary behavior and the barriers related to the capability, opportunity, and/or motivation needed to influence that target behavior (COM-B). To identify behavioral targets, we first conducted nominal groups with primary care clinicians and focus groups with patients from these settings to identify the major barriers to implementation (Phase I). The results of these interviews were used to develop an implementation strategy for increasing the uptake of the guideline in the **ambulatory care network (ACN) of New York-Presbyterian Hospital (NYP), a network of 10 primary care practices serving 120,000 patients from underserved communities in New York City** (Phase II). The Behavior Change Wheel implementation science framework was applied to develop this strategy. Health system leaders were interviewed to confirm and refine components of the strategy, including the modes of delivery. In Phase III, we conducted a 2.5-year cluster randomized trial in which we randomized 4 pairs of ACN practices (1:1) to either receive the guideline implementation strategy or to a wait-list control. Clinicians and patients were assigned to the intervention or control based on their practice's allocation. The implementation strategy was evaluated using the RE-AIM (Reach, Effectiveness, Adoption, Implementation, Maintenance) framework that combines an assessment of the reach/effectiveness of the intervention for increasing the use of out-of-office BP monitoring prior to hypertension diagnosis (primary outcome) with a mixed-methods process evaluation of the adoption, implementation, and maintenance of the implementation strategy.[20-22]

Objectives/Hypothesis

The overarching goal of this research is to conduct a cluster RCT that will rigorously evaluate the effectiveness of a theory-derived multifaceted implementation strategy at increasing out-of-office BP monitoring by patients with elevated office BP and no prior diagnosis of hypertension in accordance to updated recommendations from the USPSTF as well as other hypertension societies.

To examine in a cluster randomized trial the effectiveness of a multifaceted implementation strategy at increasing out of office BP testing in adult patients with elevated office BP but no prior diagnosis of hypertension.

Hypothesis 1a: There will be a greater increase in the percentage of eligible visits at which out-of-office BP monitoring is completed within 6 months of the visit [primary outcome] from the pre- to post-implementation period in intervention practices compared to control practices.

Hypothesis 1b: There will be a greater increase in the percentage of eligible visits at which out-of-office BP monitoring is ordered [secondary outcome] from the pre- to post-implementation period in intervention practices compared to control practices.

Hypothesis 1c: There will be a greater increase in the percentage of eligible visits at which white coat hypertension diagnosed within 6 months of the visit [secondary outcome] from the pre- to post-implementation period in intervention practices compared to control practices.

Trial Design

To accomplish this trial's objectives, we will match 4 pairs of similar primary care clinics (as per patient volume and clinician mix) and randomly assign them (1:1) to usual care or to implementation of a theory-driven multifaceted implementation strategy designed using the Behavior Change Wheel framework to increase the uptake of out-of-office BP monitoring in patients with elevated office BP but no prior diagnosis of hypertension. The implementation strategy will also be evaluated using the RE-AIM framework. Outcome assessors that extract and analyze data from the EHR will be blinded to allocation; patients and clinicians, however, will be unblinded. For intervention practices, after allowing for a 6-month implementation period in which the intervention is implemented, a cohort of eligible patients will be passively enrolled over 12 months and then followed for up to 6 months to determine out-of-office BP test completion. For control practices, a cohort of eligible patients will similarly be passively enrolled over 12 months during the same time period and then followed for up to 6 months. Corresponding groups of patients in intervention and control practices will be retrospectively enrolled across 12 months during a 12-month pre-implementation period that occurs prior to the start of the implementation of the multifaceted intervention.

Figure 1. Study Timeline

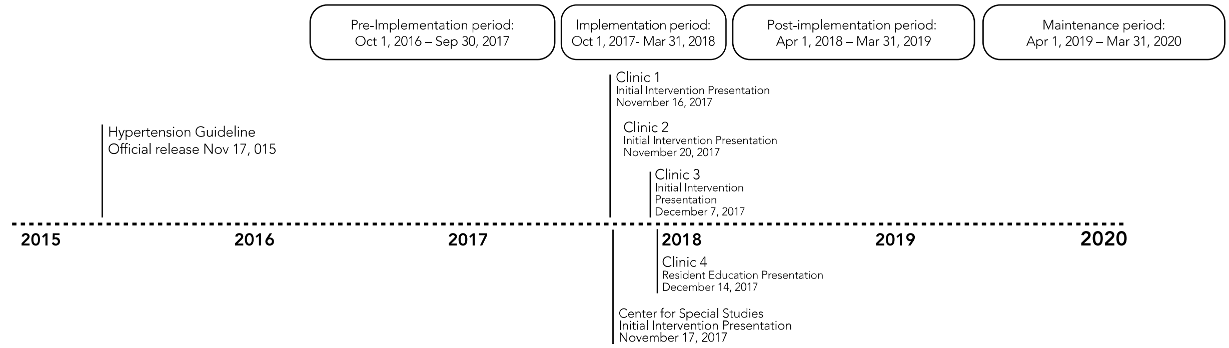

Methods

Participant, Interventions and Outcomes

Healthcare Setting:

The trial was conducted in 8 primary care practices that are part of the **Ambulatory Care Network (ACN)** of New York-Presbyterian Hospital (NYP). The ACN serves a predominantly low-income, publicly insured population with substantial numbers of Hispanic and African American patients. The catchment area includes Upper Manhattan (31% African American; 61% Hispanic descent) and Queens (11% African American; 25% Hispanic; 11% Asian/Pacific Islander). Approximately 65% of ACN patients are women, 50% Hispanic, 20% African American, and the mean age is 55 years old. The majority of patients have Medicaid or are dually eligible (Medicare and Medicaid). The primary care practices are staffed by a mix of internal medicine physicians, family practitioners, nurse practitioners, and graduate medical education (GME) trainees. During the time period of this trial, the practices used two different electronic health records (EHRs), Allscripts (Allscripts Sunrise, Allscripts, Chicago, IL) and EPIC (EPIC systems, Verona, IL), two of the largest health information technology systems in the U.S. Primary care practices that served adult patients and were part of the NYP ACN were eligible for this trial. To be included, the medical director of the practice had to agree to participate. Two practices that were primarily staffed by internal medicine residents, one affiliated with Weill Cornell and one with Columbia, were used for pilot testing components of the implementation strategy and were therefore excluded from the trial. Office BP was typically taken by medical assistants using automated devices. BP readings were manually entered into structured flowsheets in the EHR. None of the practices had protocols for systematically repeating elevated office BP readings.

Detailed Eligibility Criteria

<u>Practices</u>

Inclusion criteria:

- NYP Ambulatory Care Network primary care practice (general medicine, family medicine, nurse practitioners, comprehensive care practice for people living with

HIV, geriatrics practice) that provides primary care to adult patients, including people living with human immunodeficiency virus (HIV)

Exclusion criteria:

- Medical director declines to have the practice participate in the study
- Site for pilot testing the developing and pilot testing the implementation strategy

Patients

Eligibility criteria were selected to be consistent with those used in the 2015 USPSTF recommendations on hypertension screening

Inclusion criteria:

- Elevated blood pressure (BP) (systolic BP>=140 mmHg or diastolic BP >=90 mmHg) at a scheduled practice visit with a primary care provider from a practice that is participating in the study; if multiple BP readings were taken from a visit, then the average of the readings will be used; if sitting and standing BP readings are documented as part of an evaluation for orthostatic hypotension, then only the sitting BP readings will be averaged

Exclusion criteria (as per manual EHR review by a medically trained member of the research team):

- Prior diagnosis of hypertension
- Prior diagnosis of white-coat hypertension
- Prior evaluation for white-coat hypertension by 24-hr ABPM or HBPM
- Prescribed antihypertensive medication prior to the visit at which patient has elevated office BP without diagnosis of hypertension in the medical record
- Severely elevated office BP (systolic BP>=180 mmHg or diastolic BP>=110 mmHg); if multiple office BP readings on a given visit, then the mean was used
- Evidence of target-organ damage (chronic kidney disease as defined by diagn, cardiovascular disease)
- Manual office BP <140/90 mmHg

Intervention (Multi-Faceted Implementation Strategy) and Control Conditions

Intervention:

Following the Behavior Change Wheel framework, the theory-derived multi-faceted implementation strategy for increasing the completion of ABPM/HBPM consisted of:

1) accessible ABPM testing service located on the Columbia-campus; of note, ABPM testing was available for hypertension screening at a preventive cardiology practice on the Cornell campus, however this practice only accepted Medicare insurance and it was

not possible to order testing through a simple EHR order;

2) presentations for clinicians on how and why to order ABPM and HBPM with components designed to address motivational barriers identified in the formative qualitative work;

3) information on how to order ABPM and HBPM to clinicians via emails and other electronic communications;

4) EHR tools to facilitate ABPM and HBPM test ordering; of note, when designing the EHR tools, a hard-stop pop-up recommending out-of-office BP monitoring with associated orders was not deemed acceptable in the context of “pop-up fatigue”; a Best Practice Advisory triggered by an elevated office BP reading was created in Epic at Cornell-affiliated practices, but required a clinician to click on the BPA to get connected to resources to facilitate ABPM and HBPM; ABPM order in the preference order list was created at Columbia-affiliated practices

5) quarterly e-mail clinician feedback about extent of and outcomes of practice-level ABPM, highlighting the common identification of white-coat hypertension through ABPM testing; white-coat hypertension was identified in 40%-50% of patients referred for ABPM, depending on the practice and time period. This finding was included in feedback emails

6) brief (<30 min) nurse training on how to teach patients to conduct HBPM;

7) bilingual patient informational materials to facilitate successful ABPM/HBPM test completion.

Control:
Practices randomized to the wait-list control condition continued to screen and diagnose hypertension according to their usual practice without the benefit of the EHR tools or other clinician-directed intervention components. Patients from these practices, however, were still eligible to receive ABPM from the locally available ABPM testing center if referred by their clinicians, though no special outreach regarding the availability of this service was made as part of this study.

Overview of Study Timepoints and Measures

The key time points for the study are shown in **Figure 1**, above. Once the multifaceted implementation strategy was ready for implementation across multiple practices, medical directors were re-contacted to confirm interest and practice eligibility for participation. Practices that agree to participate were randomized to implementation versus usual care. There was a 6-month period (October 1, 2017 to March 31, 2018) during which the clinicians and staff at the practices randomized to the intervention received the implementation strategy. Over the subsequent 12 month post-implementation period (April 1, 2018 to March 31, 2019), patients that had scheduled primary care visits at which they had elevated office BP without a prior diagnosis of hypertension were passively enrolled into the trial from implementation and control

practices, with data obtained from the EHR. These patients were then passively followed for up to 6 months to assess for test completion if ordered. A separate group of patients was also passively enrolled from a 12-month pre-implementation period that took place in the year before the start of implementation (October 1, 2016 to March 31, 2017). Data for evaluating the effectiveness of implementation strategy were collected passively from the electronic health record by medically trained abstractors. Additional data to understand implementation outcomes (e.g., fidelity, acceptability) were assessed by surveying and interviewing clinicians after the post-implementation period.

Outcomes

The primary outcome was patient completion of out-of-office BP monitoring within 6 months of an eligible patient visit, either ABPM or HBPM (**Figure 1)**.

The prespecified secondary outcomes were:

(1) patient completion of ABPM testing within 6 months of an eligible patient visit.

(2) patient completion of HBPM testing within 6 months of an eligible patient visit.

(3) clinician ordered out-of-office BP monitoring, either ABPM or HBPM at the time of an eligible patient visit.

(4) clinician ordered ABPM testing at the time of an eligible patient visit.

(5) clinician ordered HBPM testing at the time of an eligible patient visit.

Medically-trained chart abstractors reviewed notes from subsequent office visits for evidence of ABPM or HBPM testing within 6 months of the visit at which tests were ordered. ABPM testing was coded as complete if sufficient awake BP readings (i.e., ≥ 10 awake BP readings) were available to estimate mean awake BP; asleep readings were not required. Determination of ABPM test completion was supplemented using data from the clinical ABPM testing service database. Outcome assessments were independently coded by a second assessor, with discrepancies resolved by consensus.

To better understand natural trends in the adoption of ABPM and HBPM in our primary care network prior to testing our implementation strategy, we additionally assessed out-of-office BP monitoring in patients with elevated office BP but no diagnosis of hypertension before (2014) and after the publication of the updated USPSTF recommendations (2016). Chart review was used to determine whether ABPM or HBPM testing were ordered and completed.

To better understand why our implementation strategy did or did not work, in May of 2019 (after the post-implementation period), we e-mailed a survey assessing barriers to ABPM and HBPM in the context of hypertension diagnosis to primary care clinicians in our ACN network. The survey assessed attitudes towards out-of-office BP monitoring as part of hypertension screening with a focus on barriers that were identified in our formative work. The survey also assessed awareness and perceived helpfulness of the individual implementation strategy components. To gain a deeper understanding of the results of this survey, we conducted interviews with high and low out-of-office BP test

ordering clinicians to understand why certain barriers remained despite our attempt to address them with our implementation strategy. To understand the sustainability of our strategy, we planned to assess ABPM and HBPM test ordering and completion in the year after the 12-month post-implementation period (i.e., the maintenance period).

Statistical Approach

Three closely related hypotheses will be used to test the effectiveness of the intervention (i.e., the multifaceted implementation strategy):

H1: The rate of out-of-office BP monitoring during the post-implementation period will be higher in practices that received the intervention than in the control practices.

H2: The rate of out-of-office BP monitoring within practices assigned to the intervention condition will be higher during the post-implementation period than during the pre-implementation period.

H3: The pre- to post-implementation period change in the likelihood of out-of-office BP monitoring will be greater in the practices that received the intervention than in the control practices.

Multilevel Poisson regression model[39,40], where level 1 is an eligible patient visit and level 2 is the practice, were used to test these hypotheses (See Statistical Analysis Plan for details). The same approach will be used to evaluate the effect of the intervention on secondary outcomes including the ordering of ABPM or HBPM testing.

Process evaluation

Quantitative data will be analyzed using descriptive statistics to assess reach, adoption, and implementation outcomes relevant to the intervention group. Additionally, to assess adoption, multi-level Poisson regression models will be used to compare the proportion of clinicians that referred at least one patient for out-of-office BP monitoring in intervention practices versus control practices. To assess implementation, multi-level linear regression models will also be used to compare clinician ratings of perceived barriers and facilitators (7-point Likert scales) to ordering out-of-office BP monitoring for guideline-eligible patients from intervention practices versus control practices; as in the primary analyses, practices will be treated as a random factor. The equivalent multi-level Poisson regression models will be used to compare intervention practices versus control practices in terms of clinician reports of whether each individual intervention component was received (yes/no). Finally, an intercept-only multi-level linear regression model will be used to estimate the average “helpfulness” of the intervention, rated on a 4-point Likert scale, for intervention practices only. Content analysis will be used to evaluate transcripts of key stakeholder interviews or focus groups, if conducted.

Sample Size and Power Considerations

Power Considerations

Our power calculations are based on data from 2014, the year prior to the update to the USPSTF hypertension screening recommendations. That year, there were 770 unique patients, with 997 eligible patient visits, seen by 169 clinicians at the 8 participating practices. Our power calculations assume this same 2014 distribution of clinicians,

patients, and eligible visits/patient for both pre- and post-implementation 12-month monitoring periods. For the effect size, we estimate that even with the release of the USPSTF guideline, there continued to be very low uptake of out-of-office BP testing to confirm hypertension diagnoses. Conservatively allowing that the average rate of out-of-office BP completion in control and intervention practices increased in the years following the publication of the USPSTF hypertension screening recommendations to as high as 5% of patient visits with incident hypertension, we estimated the power to detect a 10% increase in out-of-office BP completion rate due to the intervention (i.e., 15% vs 5%), at a two-tailed, α=0.05 significance level, for each of the 3 hypotheses described above (assuming the average out-of-office BP completion rate in control practices remained at 5%). In addition to the hypothesized out-of-office BP completion rates (5% and 15%), the power is influenced by two parameters associated with the random effects (α_{s1} and α_{s2} which capture between-practice differences/variability, also known as clustering by practice): 1) the standard deviation, σ, due to the systematic variability among practices in their log-transformed rates of out-of-office BP completion rates [σ = ln(CV), where CV, coefficient of variation is the standard deviation across practices of their out-of-office BP completion rates divided by the overall average completion rate; e.g., if CV=0.40, then the practice-specific completion rates have a standard deviation of 2% when the overall average pre-implementation completion rate equals 5% and a standard deviation of 6% when the overall average post-implementation completion rate for practices assigned to the intervention condition equals 15%], and 2) the correlation, *r*. When CV=0, there are no systematic differences among practices (i.e., no clustering by practice) and the model becomes a fixed effects, rather than a random effects, model. As CV increases, the systematic differences among the practices increases, reducing the ability (i.e., power) to generalize beyond the 8 participating practices. On the other hand, increasing levels of *r* increase the power to detect pre-implementation to post-implementation change, because it reduces the standard deviation of $\alpha_{s2} - \alpha_{s1}$.

For CV=0 and each combination of CV=0.10, 0.20, 0.30 and 0.40 with *r*=0.50, 0.60, 0.70, 0.80, and 0.90, we ran two sets of 10,000 simulations. For each simulation,

1) one practice (within each pair of matched practices) was randomly assigned to the intervention and the other to the control condition,
2) α_{s1} and α_{s2} were randomly generated from a log-bivariate normal distribution with the specified CV and *r* (thereby introducing the targeted amount of systematic variability among practices), and then,
3) using the 2014 data for each individual practice, each eligible 2014 patient-visit within that practice was randomly assigned a value of 1 (completing out-of-office BP) if a randomly generated value from a uniform(0,1) distribution was $<\exp(\alpha_{s1})$ for pre-implementation period or $<\exp(\alpha_{s2} + \beta_3 * \text{Condition})$ for post-implementation period (β_1 and β_2 were both assumed to equal zero); those visits not assigned a value of 1 were assigned a value of 0. Each 2014 patient-visit was used to generate a random observation for that practice for the pre-implementation period and a random observation for the post-implementation practice. Within a period, the generation of outcomes was constrained such that once a patient completed out-of-office BP, any subsequent eligible practice visits for that patient were ineligible for randomization.

The above-described multilevel Poisson regression model was estimated for each of the 10,000 simulated datasets, and the proportion of datasets in which the null hypothesis

was rejected, in the hypothesized direction, was an estimate of the statistical power to detect the assumed effect size. To repeat, 2 sets of 10,000 simulations were performed for each combination of CV and *r*. The study has >84% power to detect the hypothesized RR=3.0 (15% completion rate in intervention practices vs 5% in control practices; power >92% if CV≤0.30) for Hypothesis 1, the comparison of post-implementation completion rates between intervention and control practices. The study has >92% power to detect the hypothesized RR=3.0 (15% post-implementation completion rate vs 5% pre-implementation) for Hypothesis 2, the test of the change in completion rate among intervention practices only. For CV=0, the study has 84% power to detect the hypothesized Condition*Period interaction effect (Hypothesis 3), that the power drops to slightly less than 80% for low values of CV and high values of *r*, and decreases to as low as 65% for CV=0.40 and *r*=0.50.

Recruitment

Practices were recruited through communications with the ACN leaders (Director of Ambulatory Care, medical directors) that could decide whether practices should participate in the trial. These health system leaders were provided with details of the implementation strategy prior to formally enrolling in the trial.

As the intervention that the implementation strategy seeks to promote is evidence-based and considered quality improvement, a waiver of informed consent was obtained so that patients and clinicians did not need to be directly consented into the trial. For the primary analyses, we passively "enrolled" a sample of intervention group patients with elevated office BP but no diagnosis of hypertension at scheduled office visits over a 12-mo pre-implementation period, and again over a 12-mo post-implementation period after a 6-mo transition period during which the multifaceted strategy was implemented, and we then "followed" each of these patients for up to 6-months after the eligible patient visit to assess for test completion. The same procedure was used to enroll control group patients from the control practices during the pre- and post-implementation control periods.

Assignment of Interventions

Randomization and Allocation

Practices with similar properties were matched in pairs in terms of practice volume of patients and clinician-training (e.g., HIV, inclusion of trainees) mix. We then randomly assigned practices within these pairings to implementation (intervention) or usual care. Computer generated random allocation in SAS was used to assign practices. Randomization assignment became visible to the study team only after the randomization sequence was determined. Clinicians and patients were considered unblinded to treatment allocation. Personnel who extracted and analyzed quantitative data for study outcomes were blinded. To avoid contamination during the pre-implementation period, the randomization sequence and crossover date were concealed from practices until the start of the implementation period, and final intervention details, trainings, and marketing materials were only shared at the start of the implementation period.

Sequence generation

Practices were randomly assigned to one of two groups: intervention or control.

Concealment Mechanism

Practices will be assigned a study code and randomized using a computer generated random allocation. Concealment will be ensured as the randomization algorithm will run in the backend, and only the randomization assignment will be visible to the unblinded implementation team. Practice assignments will be made by a study statistician who has no role in intervention development or implementation. The study team charged with implementation will have no ability to influence allocation assignments.

Blinding

Randomization assignments were made by an unblinded member of the study team not otherwise involved in the randomization process. This individual followed the code generated by the study statistician. Thus, randomization allocation will be concealed from the study statistician (Dr. Schwartz) and Data Management Team (unless the code needs to be broken), until the point that finalized datasets are locked. Importantly, the medically trained data extractors were also blinded to group assignment by study practices.

Data Collection, Management and Analysis

Data Collection Methods

Screening and Eligibility

Screening will be accomplished in three stages. First, an EHR search of all scheduled office visits in the relevant practices during the time period of interest will be conducted. Then, the data team will organize these data and reduce the list of potentially eligible patients by programming codes that automated the exclusion according to specific criteria. These included excluding patients with non-elevated BP readings in the vital sign flowsheet and excluding patients that had prescribed BP medications on dates prior to the potentially eligible practice visit. This list of potentially eligible visits was then entered into a REDCap database. Third, two medically trained chart extractors further assessed eligibility through manual chart review using the eligibility criteria described above. Differences in eligibility determinations were resolved through consensus with a third member of the chart extraction team. All data extractors remained blinded to group assignment.

Pre-Implementation and Post-Implementation Period Assessments

Research data for the study include:

- Electronic medical record (EMR) data extraction relevant to patient characteristics of eligible patients, out-of-office BP test ordering by eligible clinicians, and out-of-office BP test completion by patients
- ABPM testing service data from the Columbia-site, to confirm no ABPM test orders or completion were missed
- Clinician survey to assess clinician characteristics (age, gender, years since completed training) as well as clinician perceptions toward barriers to out-of-office BP monitoring following the post-implementation period
- Clinician interviews to better understand clinician attitudes toward the implementation strategy components

Post-implementation period (April 1, 2018 to March 31, 2019) assessments of out-of-office BP monitoring took place by identifying eligible patient visits in the year prior to delivering the implementation strategy. Key study outcomes were then coded through manual chart extraction.

Pre-implementation period (October 1, 2016 to March 31, 2017) assessments of out-of-office BP monitoring took place by identifying eligible patient visits in the year prior to delivering the implementation strategy. Key study outcomes were then coded through manual chart extraction.

Only medically-trained individuals were eligible to manually extract data from the electronic medical record. These individuals were blinded to group assignment.

Data Management

Data Entry, Security and Storage

A dedicated, HIPAA-compliant, web-based data entry system called REDCap will be used for this study. Data coding will occur separately at Cornell and Columbia. Cornell

data will be de-identified before being transferred to Columbia. All data are stored in a secure server at Columbia University Irving Medical Center, and automatically backed up according to an established regular schedule.

Data Discrepancies and Resolutions

Data discrepancies in coding eligibility, patient characteristics, or eligibility will be resolved by consensus. The study statistician will send a list of errors and discrepancies with detailed descriptions to data coders to reconcile. All sites are required to address all items in the report by checking EHR to correct any inconsistency, or by declaring the item as permanently missing. The local sites' study personnel will be responsible for updating and correcting the data entry in the web-based system.

Statistical Methods

All analyses will use the principle of intention-to-treat. Baseline patient characteristics will be examined as means (standard deviation) or percentages by randomization assignment and period (pre-implementation versus post-implementation) to assess for a balanced allocation. Also, clinician characteristics will be compared across group assignment.

Effectiveness of intervention. The relative change, from the 12-month pre-implementation period to the 12-month post-implementation period, in the proportion of patient visits after which guideline-eligible patients complete out-of-office BP testing (either ABPM or HBPM) will be the primary outcome measured at the practice level. This change will be estimated using a multilevel Poisson regression model in which level 1 is the eligible patient and level 2 is the practice.[1,2] There will be two observations for each of the 8 practices, one for the pre-implementation and one for the post-implementation 12-month period, and each observation will include:

1) the outcome: the number of patients who completed out-of-office BP testing within six months of an eligible visit that occurred within the given 12-month period,
2) the total number of unique eligible patient-visits during the given 12-month period (used as an "offset" variable, after log-transformation, in the Poisson regression), and
3) two binary predictors, Condition (0=control site, 1=intervention site) and Period (0=pre-implementation, 1=post-implementation).

The model will include two correlated random intercepts, one to capture site-specific differences (i.e., clustering by site) during the pre-implementation period and one for the post-implementation period. We considered a much more complex model that would include random effects for each individual physician, but recent work suggests that it is sufficient to model only clustering at the highest level of analysis at which clustering is assumed to occur.[3] Using a log link function, we will estimate the following model:

$$\ln(OOO\_BP_{sp}) = \alpha_{sp} + \beta_1{*}Condition_s + \beta_2{*}Period_p + \beta_3{*}Condition_s{*}Period_p$$

Where, s indexes practice sites, s = 1 to 8,

p equals 0 for pre-implementation period, 1 for post-implementation period,

OOO_BP_{sp} equals the number of out-of-office BP assessments at site s during period p,

α_{s1} equals the site specific effect for the pre-implementation period,

α_{s2} equals the site specific effect for the post-implementation period, and
α_{s1} and α_{s2} are assumed to have equal standard deviations, σ, and a bivariate normal distribution with correlation *r*.

This analysis will be used to test three closely related hypotheses:

1) The rate of out-of-office BP completion during the post-implementation period will be higher in the practices that received the intervention than in the control practices. $\text{Exp}(\beta_1 + \beta_3)$ equals the relative risk (RR) of out-of-office BP assessment in intervention compared to control practices during the post-implementation period, and testing the hypothesis that $\beta_1 + \beta_3 = 0$ is equivalent to testing the hypothesis that this RR=1.0.
2) The rate of out-of-office BP completion within practices assigned to the intervention condition will be higher during the post-implementation period than during the pre-implementation period. $\text{Exp}(\beta_2 + \beta_3)$ equals the RR of out-of-office BP assessment during the post-implementation period compared to the pre-implementation period, within the practices assigned to the intervention condition, and testing the hypothesis that $\beta_2 + \beta_3 = 0$ is equivalent to testing the hypothesis that this RR=1.0.
3) The pre- to post- change in the likelihood of out-of-office BP testing will be greater in the practices that received the intervention than in the control practices. B_3, the coefficient of the interaction term, reflects the extent to which the Pre- to Post- change in the likelihood of out-of-office BP testing is greater in the practices that received the intervention than in the control practices. More specifically, if RR_0 equals the relative risk of out-of-office BP completion during the post-implementation period compared to the pre-implementation period in control practices and RR_1 equals the corresponding relative risk in intervention practices, then $\exp(\beta_3)$ equals RR_1/RR_0, and testing the hypothesis that $\exp(\beta_3)$ =0 is equivalent to testing the hypothesis that $RR_1/RR_0 = 1$.

Although we anticipate that the matching of practices will have promoted balance between those practices assigned to the intervention and control conditions, we will not incorporate the matching into the analysis. The same approach will be used to evaluate the effect of the intervention on secondary outcomes including the rate of ABPM or HBPM ordering.

Accounting for Clustering

Overall, we incorporated between-site variability in out-of-office BP completion rates at both baseline and follow-up (with variability equal to 0, 10%, 20%, 30% or 40% of the average rate), and a correlation between the site-specific rates of r=0.50, 0.60, 0.70, 0.80, or 0.90. The fact that r was assumed to be <1.00 means that unlike most power analyses, we not only incorporated between-site variability in average levels, but we also incorporated between-site variability in change from baseline to follow-up. Thus, GEE cannot be used to perform the analysis because there are two random effects which can be viewed, equivalently, either as Site-specific differences (clustering) at baseline and Site-specific differences at follow-up, OR as Site-specific differences at baseline and Site-specific differences in change from baseline to follow-up.

Sensitivity Analysis

The USPSTF hypertension screening guidelines do not specify how to determine whether office BP is elevated, though they do note that office BP has variability, and averaging office BP or seeing a pattern of elevated office BP across two or more office visits might be appropriate for determining elevated office BP prior to ordering out-of-office BP monitoring. Accordingly, we will perform sensitivity analyses by assessing outcomes in the subgroup of patients who had elevated BP readings in the prior scheduled office visit as well as at the eligible office visit.

To better understand differences in implementation and usual care within individual practices, we will also explore outcomes at the practice level.

References

1. Snijders T, Bosker R. Multilevel Analysis: An Introduction to Basic and Advanced Multilevel Modeling (Second Edition). London: Sage Publications; 2012.
2. Goldstein H. Multilevel Statistical Models. West Sussex: John Wiley & Sons Ltd.; 2011.
3. Murray DM, Hannan PJ, Baker WL. A Monte Carlo study of alternative responses to intraclass correlation in community trials. Is it ever possible to avoid Cornfield's penalties? *Eval Rev*. 1996;20:313-337.

Data Monitoring

Since EMBRACE represents the implementation of a strategy designed to increase the uptake of an evidence-based practice, since the delivery of this strategy was deemed to be associated with minimal risks, and since the trial will not involve prospective enrollment of patient or providers and clinical outcomes will not be prospectively assessed, the randomized trial is not a “clinical trial” according to the Public Health Service definition and does not require a Data Safety and Monitoring Board (DSMB).

Harms

While no serious harms were expected, given that intervention patients could conceivable be more likely to experience harms from ABPM testing in terms of severe bruising or other unexpected adverse event from ABPM testing, we did query our ABPM testing service database and ask the medically-trained chart extractors to note if there were any unanticipated AEs that must be reported to the IRB according to standard guidelines.

Any AE or unanticipated problem will be identified, responded to, recorded by the site investigator, who will in turn ensure that the information is passed on. If the AE or problem is unexpected, related to study involvement, and puts the participant at increased risk, it will be reported to the local IRB immediately, in accordance with local policies. These events will also be reported to Columbia University IRB.

Auditing

Routine audits of data completion and timeliness will be overseen by the study statistician in conjunction with the Quantitative Data Committee.

Ethics and Dissemination

Research Ethics Approval

This protocol has been approved by Columbia University Irving Medical Center's and Weill Cornell Medicine's Institutional Review Board (IRB) with respect to scientific content and compliance with applicable research and human subjects regulations. Each study site must submit verification of IRB approval to the Columbia site prior to the initiation of study activities.

Subsequent to initial review and approval, Columbia University's and Weill Cornell's IRB will review the protocol at least annually. The overall PI and site PIs will make safety and progress reports to the IRBs are completed at least annually. These reports will include the total number of participants enrolled, reports of any adverse events, and any other requested reports.

Protocol Amendments

Any modifications to the protocol which may impact on the conduct of the study, potential benefit of the patient or may affect patient safety, including changes of study objectives, study design, patient population, sample sizes, study procedures, or significant administrative aspects will require a formal amendment to the protocol. Such amendment will be agreed upon by the overall PI, site PIs and co-investigators, and approved by the responsible IRB prior to implementation and notified to the health authorities in accordance with local regulations.

Administrative changes of the protocol are minor corrections and/or clarifications that have no effect on the way the study is to be conducted. These administrative changes will be agreed upon by the overall PI and site PIs, and will be documented in a memorandum. The responsible IRBs may be notified of administrative changes at the discretion of the PIs.

Informed Consent

The protocol and informed consent procedures will be approved by the Institutional Review Boards at each participating institution. Given the minimal risk nature of the study, a waiver of informed consent was obtained for extracting patient data from medical records in conjunction with assessing primary and secondary outcomes of the effectiveness of this study's implementation strategy. A signed informed consent was not required for clinician surveys as again, these surveys were optional, voluntary, and conferred no risk. Further, completion of the survey was deemed to indicate consent.

A verbal informed consent was obtained prior to telephone interviews with clinicians eligible for this trial to gain a deeper understanding of attitudes toward the implementation strategy after the post-implementation period. At the time of enrollment, the staff member conducting these interviews would give a complete description of the study to the participant in clear, easy-to-understand language. After reading and understanding the consent and the procedures, those who choose to participate will give their verbal consent to proceed with the interview.

All staff involved in this study will have completed and passed GCP and HIPAA training, and will have been provided with materials and instruction in the proper and ethical manner in which consent should be obtained. When telephone consent is obtained, it will comply with all GCP and HIPAA regulations, and be IRB-approved.

Confidentiality

As part of the process involved in obtaining written informed consent, all participants will be reminded that their responses are confidential and that they may refuse to participate in the study or withdraw at any time without explanation, and further, that such an action will in no way affect their future interactions with their participating Medical Center.

To ensure confidentiality, all study-related information will be stored securely at the study sites. When paper records are obtained, those containing names or other personal identifiers, such as locator forms, medical records, and informed consent forms, will be stored separately from study records identified by participant ID number. Forms, lists, logbooks, appointment books, and any other listings that link participant ID numbers to other identifying information will be stored in a separate, locked file in an area with limited access. All databases will be secured with password-protected access systems. Datasets for analysis will be associated with an individual participant only by an assigned identification number.

All staff will have all relevant IRB and HIPAA training in the protection of human subject participants (Good Clinical Practice).

Declaration of Interests

All investigative staff has reported any conflicts of interest to their local IRB as part of the protocol approval process.

Access to data

Only authorized personnel, those who have official status as part of the authorized research team, will have access to any records containing identifiable participant data. Study personnel at local sites will only have access to their own site’s data, and will be limited by their “user-roles” (i.e. Weill Cornell study personnel will only have access to Weill Cornell patient data, blinded data extractors will not have access to group allocation).The Quantitative Data Committee will oversee the intra-study data sharing process.

Dissemination policy

Study results will be posted at ClinicalTrials.Gov within or as soon after one year of study completion as is possible. A Publications and Disseminations Committee will be formed, and this committee will then create a policy/guideline for authorship and review process of manuscripts and abstracts for publication or conference presentations.

Title of Project:

Effectiveness of a Multifaceted Implementation Strategy for Increasing Out-of-Office Blood Pressure Monitoring during Hypertension Screening in Primary Care: The EMBRACE Cluster Randomized Trial

STATISTICAL ANALYSIS PLAN

V2 Last updated August 10, 2023

Principal Investigator

Ian Kronish, MD, MPH
Associate Professor of Medicine
Associate Director, Center for Behavioral Cardiovascular Health
Co-Director, Columbia Hypertension Center
Division of General Medicine
Columbia University Irving Medical Center

Summary of Changes to the Statistical Analysis Plan

1. Clarified that level 1 of the Poisson regression model used to compare pre- to post-implementation outcomes in intervention vs control clinics is the “patient visit”, and not “patient”.

2. Change the wording from pre- and post-intervention periods to pre- and post-implementation periods to be consistent with wording used in the study protocol

3. Clarified that we included total number of eligible visits in our modeling.

Statistical Approach

Effectiveness of intervention. The primary outcome, assessed for each eligible patient visit, will be the completion (yes or no) of out-of-office BP testing (either ABPM or HBPM) within 6 months of the visit. The secondary outcome is whether the provider ordered out-of-office BP testing (either ABPM or HBPM) at the time of the eligible visit. The data will be aggregated to the practice level, separately for pre-implementation eligible visits and post-implementation eligible visits to determine the total number of eligible visits, and the number and proportion of eligible visits with the guideline-recommended outcome for each practice and period (12-month pre-implementation period and 12-month post-implementation period). Our interest is in the relative change, from the 12-month pre-implementation period to the 12-month post-implementation period, in the proportion of eligible patient visits for which the patient completes out-of-office BP testing (either ABPM or HBPM) in those clinics randomly assigned to the intervention condition and whether this differs from the relative change in clinics assigned to the control condition. This change will be estimated using a multilevel Poisson regression model in which level 1 is the eligible patient visit and level 2 is the practice.[1,2] After aggregation, there will be two observations for each of the 8 practices, one for the pre-implementation and one for the post-implementation 12-month period, and each observation will include:

1) the number of patients who completed out-of-office BP testing within six months of an eligible visit that occurred within the given 12-month period (OOO_BP),
2) the total number of unique eligible patient visits during the given 12-month period (N_visits; used as an "offset" variable, after log-transformation, in the Poisson regression),
3) two binary predictors, Condition (0=control practice, 1=intervention practice) and Period (0=pre-implementation, 1=post-implementation), and
4) a Clinic_ID variable that uniquely identifies each of the 8 clinics.

The model will include two correlated random intercepts, one to capture site-specific differences (i.e., clustering by site) during the pre-implementation period and one for the post-implementation period. We considered a much more complex model that would include random effects for each individual physician or for each patient (given that a small number had more than one eligible visit), but recent work suggests that it is sufficient to model only clustering at the highest level of analysis at which clustering is assumed to occur.[3] Using a log link function, we will estimate the following model:

$$\ln(OOO\_BP_{sp} / N\_visits_{sp}) = \alpha_{sp} + \beta_1{*}Condition_s + \beta_2{*}Period_p + \beta_3{*}Condition_s{*}Period_p$$

Where, *s* indexes practice sites, *s* = 1 to 8,

p equals 0 for pre-implementation period, 1 for post-implementation period,
OOO_BP_{sp} equals the number of out-of-office BP assessments at site s during period p,
N_visits_{sp} equals the total number of eligible visits at site s during period p,
α_{s1} equals the site-specific effect for the pre-implementation period (a random effect),
α_{s2} equals the site-specific effect for the post-implementation period (a second random effect), and
α_{s1} and α_{s2} are assumed to have equal standard deviations, σ, and a bivariate normal distribution with correlation *r*.

This analysis will be used to test three closely related hypotheses:

1) The rate of out-of-office BP completion during the post-implementation period will be higher in the practices that received the intervention than in the control practices. $\text{Exp}(\beta_1 + \beta_3)$ equals the relative risk (RR) of out-of-office BP assessment in intervention compared to control practices during the post-implementation period, and testing the hypothesis that $\beta_1 + \beta_3 = 0$ is equivalent to testing the hypothesis that this RR=1.0 (Hyp 1).
2) The rate of out-of-office BP completion within practices assigned to the intervention condition

will be higher during the post-implementation period than during the pre-implementation period. Exp($\beta_2 + \beta_3$) equals the RR of out-of-office BP assessment during the post-implementation period compared to the pre-implementation period, within the practices assigned to the intervention condition, and testing the hypothesis that $\beta_2 + \beta_3 = 0$ is equivalent to testing the hypothesis that this RR=1.0 (Hyp 2).

3) The pre- to post- change in the likelihood of out-of-office BP testing will be greater in the practices that received the intervention than in the control practices. B_3, the coefficient of the interaction term, reflects the extent to which the Pre- to Post- change in the likelihood of out-of-office BP testing is greater in the practices that received the intervention than in the control practices. More specifically, if RR_0 equals the relative risk of out-of-office BP completion during the post-implementation period compared to the pre-implementation period in control practices and RR_1 equals the corresponding relative risk in intervention practices, then exp(β_3) equals RR_1/RR_0, and testing the hypothesis that exp(β_3) =0 is equivalent to testing the hypothesis that $RR_1/RR_0 = 1$ (Hyp 3).

Although we anticipate that the matching of practices will have promoted balance between those practices assigned to the intervention and control conditions, we will not incorporate the matching into the analysis. The same approach will be used to evaluate the effect of the intervention on secondary outcomes including provider ordering of ABPM or HBPM at the time of an eligible visit.

Power Considerations

Our power calculations are based on data from 2014, the year prior to the update to the USPSTF hypertension screening recommendations. That year, there were 770 unique patients, with 997 eligible patient visits, seen by 169 clinicians at the 8 participating practices. Our power calculations assume this same 2014 distribution of clinicians, patients, and eligible visits/patient for both pre- and post-implementation 12-month monitoring periods. For the effect size, we estimate that even with the release of the USPSTF guideline, there continued to be very low uptake of out-of-office BP testing to confirm hypertension diagnoses. Conservatively allowing that the average rate of out-of-office BP completion in control and intervention practices increased in the years following the publication of the USPSTF hypertension screening recommendations to as high as 5% of patient visits with incident hypertension, we estimated the power to detect a 10% increase in out-of-office BP completion rate due to the intervention (i.e., 15% vs 5%), at a two-tailed, α=0.05 significance level, for each of the 3 hypotheses described above (assuming the average out-of-office BP completion rate in control practices remained at 5%). In addition to the hypothesized out-of-office BP completion rates (5% and 15%), the power is influenced by two parameters associated with the random effects (α_{s1} and α_{s2} which capture between-practice differences/variability, also known as clustering by practice): 1) the standard deviation, σ, due to the systematic variability among practices in their log-transformed rates of out-of-office BP completion rates [σ = ln(CV), where CV, coefficient of variation is the standard deviation across practices of their out-of-office BP completion rates divided by the overall average completion rate; e.g., if CV=0.40, then the practice-specific completion rates have a standard deviation of 2% when the overall average pre-implementation completion rate equals 5% and a standard deviation of 6% when the overall average post-implementation completion rate for practices assigned to the intervention condition equals 15%], and 2) the correlation, *r*. When CV=0, there are no systematic differences among practices (i.e., no clustering by practice) and the model becomes a fixed effects, rather than a random effects, model. As CV increases, the systematic differences among the practices increases, reducing the ability (i.e., power) to generalize beyond the 8 participating practices. On the other hand, increasing levels of *r* increase the power to detect pre-implementation to post-implementation change, because it reduces the standard deviation of $\alpha_{s2} - \alpha_{s1}$.

For CV=0 and each combination of CV=0.10, 0.20, 0.30 and 0.40 with *r*=0.50, 0.60, 0.70, 0.80, and 0.90, we ran two sets of 10,000 simulations. For each simulation,

1) one practice (within each pair of matched practices) was randomly assigned to the intervention and the other to the control condition,
2) α_{s1} and α_{s2} were randomly generated from a log-bivariate normal distribution with the specified CV and *r* (thereby introducing the targeted amount of systematic variability among practices), and then,
3) using the 2014 data for each individual practice, each eligible 2014 patient-visit within that practice was randomly assigned a value of 1 (completing out-of-office BP) if a randomly generated value from a uniform(0,1) distribution was $<\exp(\alpha_{s1})$ for pre-implementation period or $<\exp(\alpha_{s2} + \beta_3 * \text{Condition})$ for post-implementation period (β_1 and β_2 were both assumed to equal zero); those visits not assigned a value of 1 were assigned a value of 0. Each 2014 patient-visit was used to generate a random observation for that practice for the pre-implementation period and a random observation for the post-implementation practice. Within a period, the generation of outcomes was constrained such that once a patient completed out-of-office BP, any subsequent eligible practice visits for that patient were ineligible for randomization.

The above-described multilevel Poisson regression model was estimated for each of the 10,000 simulated datasets, and the proportion of datasets in which the null hypothesis was rejected, in the hypothesized direction, was an estimate of the statistical power to detect the assumed effect size. To repeat, 2 sets of 10,000 simulations were performed for each combination of CV and *r*. The results are shown in the **Figure**. In each panel, the solid red horizontal line shows the power if CV=0. Panel A shows that for all considered values of CV and *r*, the study has >84% power to detect the hypothesized RR=3.0 (15% completion rate in intervention practices vs 5% in control practices; power >92% if CV≤0.30) for Hypothesis 1, the comparison of post-implementation completion rates between intervention and control practices. Panel B shows that for all considered values of CV and *r*, the study has >92% power to detect the hypothesized RR=3.0 (15% post-implementation completion rate vs 5% pre-implementation) for Hypothesis 2, the test of the change in completion rate among intervention practices only. Finally, Panel C shows that for CV=0, the study has 84% power to detect the hypothesized Condition*Period interaction effect (Hypothesis 3), that the power drops to slightly less than 80% for low values of CV and high values of *r*, and decreases to as low as 65% for CV=0.40 and *r*=0.50.

Accounting for Clustering

Overall, we incorporated between-site variability in out-of-office BP completion rates at both baseline and follow-up (with variability equal to 0, 10%, 20%, 30% or 40% of the average rate), and a correlation between the pre-implementation and post-implementation site-specific rates of r=0.50, 0.60, 0.70, 0.80, or 0.90. The fact that r was assumed to be <1.00 means that unlike most power analyses, we not only incorporated between-site variability in average levels, but we also incorporated between-site variability in change from baseline to follow-up. Thus, GEE cannot be used to perform the analysis because there are two random effects which can be viewed, equivalently, either as site-specific differences (clustering) at baseline and site-specific differences at follow-up, OR as site-specific differences at baseline and site-specific differences in change from baseline to follow-up.

References

1. Snijders T, Bosker R. Multilevel Analysis: An Introduction to Basic and Advanced Multilevel Modeling (Second Edition). London: Sage Publications; 2012.
2. Goldstein H. Multilevel Statistical Models. West Sussex: John Wiley & Sons Ltd.; 2011.

3. Murray DM, Hannan PJ, Baker WL. A Monte Carlo study of alternative responses to intraclass correlation in community trials. Is it ever possible to avoid Cornfield's penalties? *Eval Rev*. 1996;20:313-337.

Figure. Power estimates for three hypothesized tests of the intervention's effect on out-of-office blood pressure completion rates.

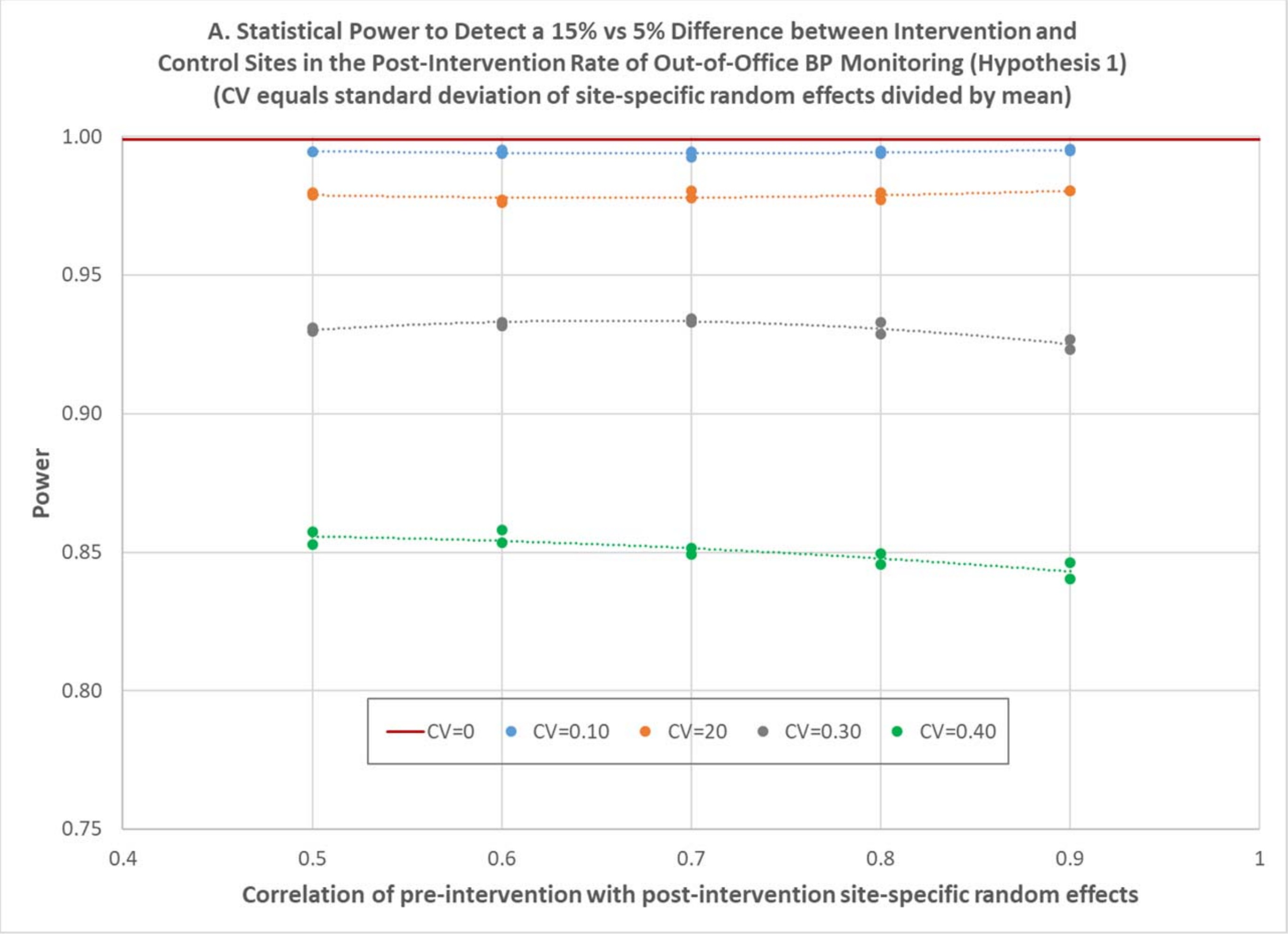

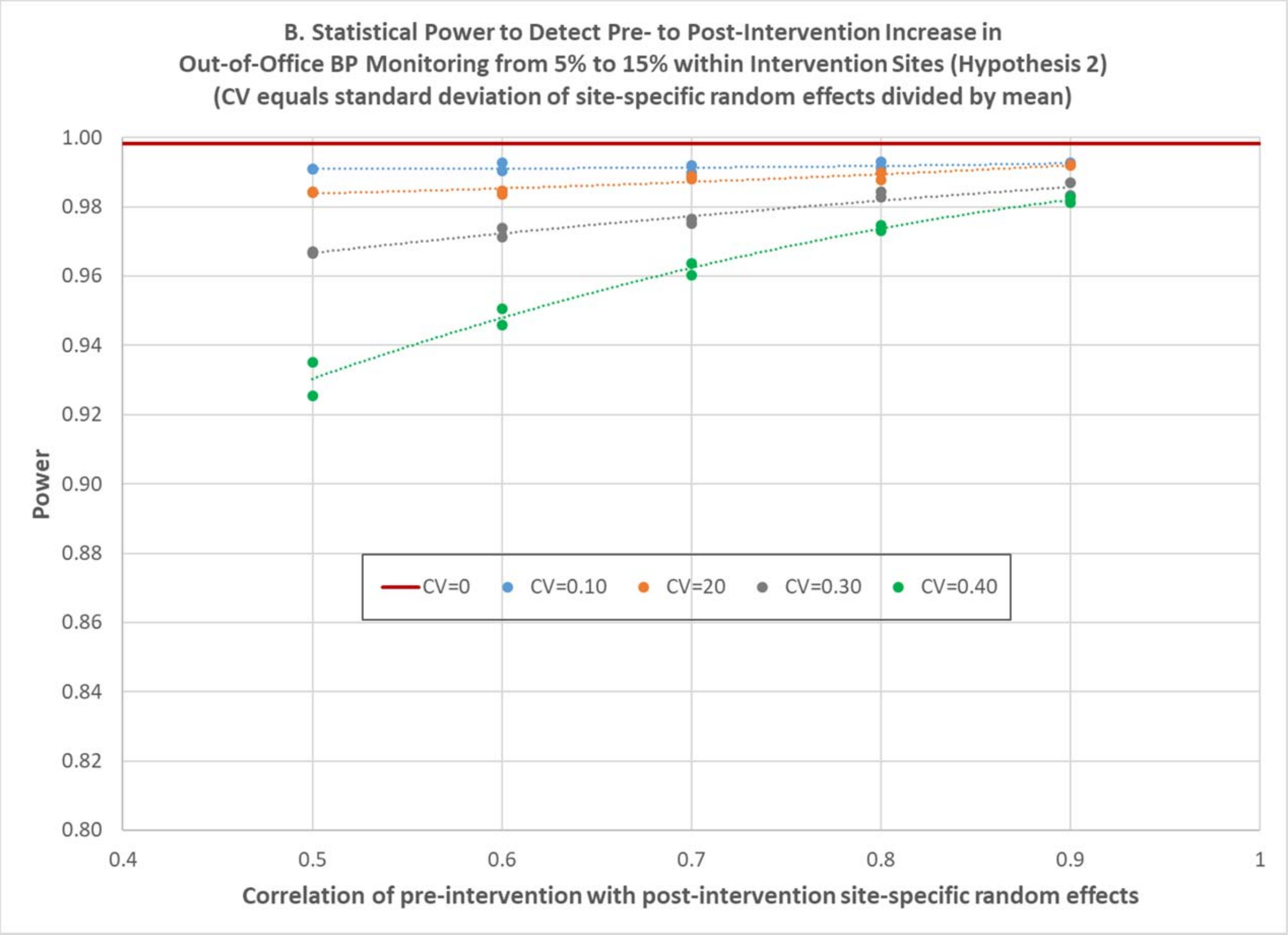
B. Statistical Power to Detect Pre- to Post-Intervention Increase in
Out-of-Office BP Monitoring from 5% to 15% within Intervention Sites (Hypothesis 2)
(CV equals standard deviation of site-specific random effects divided by mean)
Power
1.00
0.98
0.96
0.94
0.92
0.90
0.88
0.86
0.84
0.82
0.80
CV=0
CV=0.10
CV=20
CV=0.30
CV=0.40
0.4
0.5
0.6
0.7
0.8
0.9
1
Correlation of pre-intervention with post-intervention site-specific random effects

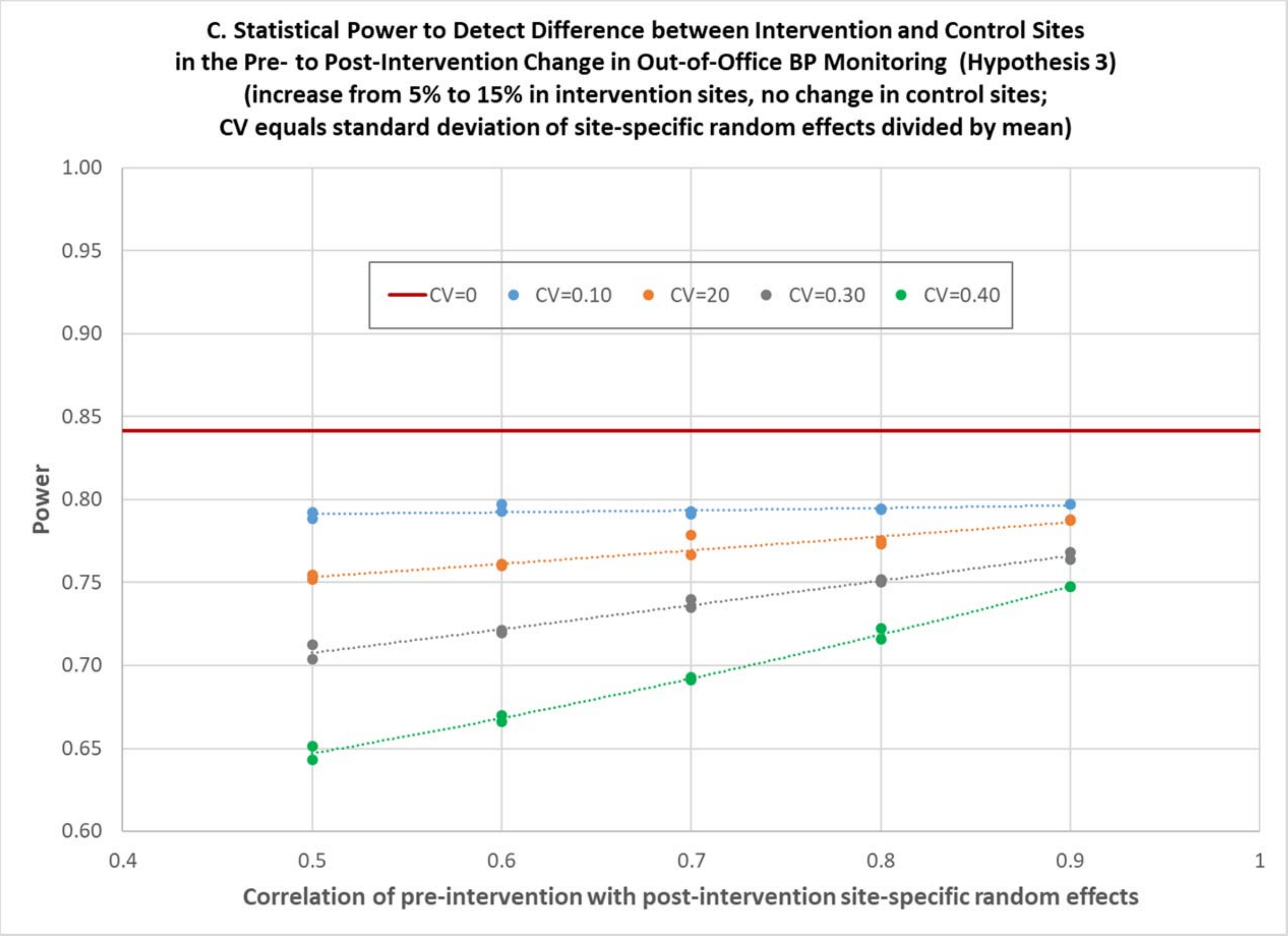
C. Statistical Power to Detect Difference between Intervention and Control Sites
in the Pre- to Post-Intervention Change in Out-of-Office BP Monitoring (Hypothesis 3)
(increase from 5% to 15% in intervention sites, no change in control sites;
CV equals standard deviation of site-specific random effects divided by mean)
CV=0
CV=0.10
CV=20
CV=0.30
CV=0.40
Power
1.00
0.95
0.90
0.85
0.80
0.75
0.70
0.65
0.60
0.4
0.5
0.6
0.7
0.8
0.9
1
Correlation of pre-intervention with post-intervention site-specific random effects
