# Supplementary material for: A Multifaceted Implementation Strategy to Increase Out-of-Office Blood Pressure Monitoring: The EMBRACE Cluster Randomized Clinical Trial
Source: JAMA Netw Open. 2023 Sep 25;6(9):e2334646. doi: 10.1001/jamanetworkopen.2023.34646 (PMC10520739; doi:10.1001/jamanetworkopen.2023.34646)
Supplement: Supplement 2. — eTable 1. Description of Implementation Strategy Components eTable 2. Clinician Characteristics According to Group Assignment eTable 3. Relative Risks (95% Confidence Interval) of Ordering and Completing Out-of-Office Blood Pressure Monitoring in the Post- vs Pre-Implementation Periods, Adjusting for Age and Sex [file jamanetwopen-e2334646-s002.pdf]

Supplemental Online Content

Kronish IM, Phillips E, Alcantara C, et al. A multifaceted implementation strategy to increase out-of-office blood pressure monitoring: the EMBRACE cluster randomized trial. *JAMA Netw Open.* 2023;6(9):e2334646.
doi:10.1001/jamanetworkopen.2023.34646

eTable 1. Description of Implementation Strategy Components

eTable 2. Clinician Characteristics According to Group Assignment

eTable 3. Relative Risks (95% Confidence Interval) of Ordering and Completing Out-of-Office Blood Pressure Monitoring in the Post- vs Pre-Implementation Periods, Adjusting for Age and Sex

This supplemental material has been provided by the authors to give readers additional information about their work.

© 2023 Kronish IM et al. *JAMA Network Open.*

eTable 1. Description of Implementation Strategy Components

Intervention Level	Intervention Component	Mode of Delivery, Timing, Frequency	Tailoring
Health System/Clinician	Access to ABPM for primary care patients	Available for referrals at the start of the implementation period	CUIMC practices: Access was through an ABPM service that accepted all insurances and included a self-pay option.<br><br>WCM practices: Access was through referral to a preventive cardiology practice that accepted Medicare and commercial insurance, only; WCM could also refer patients to the CUIMC ABPM service.
	EHR tools that promote out-of-office BP test ordering	Embedded in the EHR at the start of the implementation period	CUIMC practices: orders for ABPM and HBPM devices were embedded in a frequently used preference order set.<br><br>WCM practices: Epic Best Practice Advisory (BPA) triggered in patients with elevated office BP, but with no hard stops. If clicked, the BPA showed 1) the USPSTF hypertension screening recommendation; 2) orders for ABPM at the WCM-affiliated preventive cardiology practice, as well as instructions for ordering ABPM at CUIMC's testing service.
	Education about rationale for out-of-office BP monitoring and information on how to conduct ABPM and HBPM	One-time in-person presentation to clinicians, delivered by implementation team physician during implementation period	Information on how to order out-of-office BP monitoring was tailored for CUIMC- and WCM-affiliated practices according to differences in access to ABPM service and EHR tools
	Reminders of how to order out-of-office BP monitoring	E-mails sent by medical directors every 3 months during the post-implementation	None

© 2023 Kronish IM et al. *JAMA Network Open.*

		period	
	Feedback on the number of ABPM referrals and white coat hypertension diagnoses at the practice level	E-mails including simple bar graphs sent by medical directors every 3 months during post-implementation period	None
Health System/Nurse	Training registered nurses to teach patients how to correctly conduct HBPM	One-time in-person 30 minute training delivered by implementation team physician during the implementation period	None
Patient	Patient information handouts relevant to correctly completing ABPM and HBPM, in English and Spanish	Printable handouts, distributed to practices via email at the start of the implementation period	CUIMC practices: printable materials available from a web-based resource guide<br><br>WCM practices: printable ABPM and HBPM patient handouts linked to BPA when ABPM or HBPM were ordered in Epic

Abbreviations: ABPM, ambulatory blood pressure monitoring; HBPM, home blood pressure monitoring; BP, blood pressure; EHR, electronic health record; CUIMC, Columbia University Irving Medical Center; WCM, Weill Cornell Medicine;

© 2023 Kronish IM et al. *JAMA Network Open.*

eTable 2. Clinician Characteristics According to Group Assignment*

	Intervention (N= 4 practices)		Usual Care (N=4 practices)	
**Clinician Characteristic**	**Pre-Implementation (N=72 clinicians)**	**Post-Implementation (N=74 clinicians)**	**Pre-Implementation (N=41 clinicians)**	**Post-Implementation (N=38 clinicians)**
Age, mean (SD)	42.9 (13.1)	38.9 (12.3)	45.6 (13.4)	46.5 (15.7)
N missing	43	37	30	25
Female sex, N (%)	30 (71.4%)	30 (62.2%)	20 (90.9%)	18 (69.2%)
N missing	30	25	19	12
Training, N (%)				
Internal medicine	24 (42.1%)	19 (30.6%)	8 (32.0%)	8 (36.4%)
Geriatrics	0 (0.0%)	2 (3.2%)	14 (56.0%)	12 (54.5%)
HIV	11 (19.3%)	10 (16.1%)	3 (12.0%)	2 (9.1%)
Family medicine	22 (38.6%)	31 (50.0%)	0 (0.0%)	0 (0.0%)
N missing	15	12	16	16
Level of training, N (%)				
Attending	23 (50.0%)	22 (44.9%)	17 (68.0%)	16 (66.7%)
Resident/Fellow	19 (41.3%)	24 (49.0%)	5 (20.0%)	6 (25.0%)
Nurse practitioner	3 (6.5%)	3 (6.1%)	3 (12.0%)	2 (8.3%)
Other	1 (2.2%)	0 (0.0%)	0 (0.0%)	0 (0.0%)
N missing	26	25	16	14
Years since completed training, median (IQR)	10 (3, 20)	3 (1, 17)	9 (3, 23)	14 (3, 31)
N missing	43	36	30	25

*Abbreviations: HIV, human immunodeficiency virus

© 2023 Kronish IM et al. *JAMA Network Open.*

eTable 3. Relative Risks (95% Confidence Interval) of Ordering and Completing Out-of-Office Blood Pressure Monitoring in the Post- vs Pre-Implementation Periods, Adjusting for Age and Sex*

Outcome	Post- vs Pre-Implementation, Intervention	P-Value	Post- vs Pre-Implementation, Usual Care	P-Value	Post- vs Pre-Implementation in Intervention vs Usual Care	P-Value
ABPM or HBPM completed by patient	9.94 (2.23-44.40)	0.009	0.93 (0.28-3.11)	0.89	10.70 (1.93-59.23)	0.01
HBPM completed by patient	3.36 (0.65-17.38)	0.12	0.91 (0.27-3.05)	0.86	3.69 (0.60-22.82)	0.14
ABPM completed by patient†	N/A		N/A		N/A	
ABPM or HBPM ordered by clinician	2.75 (1.29-5.85)	0.02	1.22 (0.50-2.98)	0.60	2.25 (0.79-6.37)	0.12
HBPM ordered by clinician	1.04 (0.41-2.60)	0.92	1.19 (0.49-2.91)	0.65	0.87 (0.28-2.73)	0.80
ABPM ordered by clinician†	N/A		N/A		N/A	

*Abbreviations: ABPM, ambulatory blood pressure monitoring; HBPM, home blood pressure monitoring

†Not analyzed as no ABPM completed or ordered in pre-implementation periods in either arm nor in the post-implementation period in the usual care arm

© 2023 Kronish IM et al. *JAMA Network Open.*
