# Supplementary material for: A Multifaceted Implementation Strategy to Increase Out-of-Office Blood Pressure Monitoring: The EMBRACE Cluster Randomized Clinical Trial
Source: JAMA Netw Open. 2023 Sep 25;6(9):e2334646. doi: 10.1001/jamanetworkopen.2023.34646 (PMC10520739; doi:10.1001/jamanetworkopen.2023.34646)
Supplement: Supplement 3. — Data Sharing Statement [file jamanetwopen-e2334646-s003.pdf]

Data Sharing Statement

Data
Data available: Yes
Data types: Deidentified participant data, Data dictionary
How to access data: as5068@cumc.columbia.edu
When available: With publication

Supporting Documents
Document types: Statistical/analytic code
How to access documents: as5068@cumc.columbia.edu>
When available: With publication

Additional Information
Who can access the data: Researchers whose proposed use of the data is approved.
Types of analyses: For a specified purpose
Mechanisms of data availability: With investigator support
Any additional restrictions: None
